# Chemotion ELN: an Open Source electronic lab notebook for chemists in academia

**DOI:** 10.1186/s13321-017-0240-0

**Published:** 2017-09-25

**Authors:** Pierre Tremouilhac, An Nguyen, Yu-Chieh Huang, Serhii Kotov, Dominic Sebastian Lütjohann, Florian Hübsch, Nicole Jung, Stefan Bräse

**Affiliations:** 1 Institute of Toxicology and Genetics, Karlsruhe Institute of Technology, Hermann-von-Helmholtz-Platz 1, 76344 Eggenstein-Leopoldshafen, Germany; 2 Institute of Organic Chemistry, Karlsruhe Institute of Technology, Fritz-Haber-Weg 6, 76131 Karlsruhe, Germany; 3 Ninja-Concept GmbH, Haid-und-Neu-Straße 18, 76131 Karlsruhe, Germany; 4 Cubuslab GmbH, Lange Straße 2, 76199 Karlsruhe, Germany

**Keywords:** Electronic lab notebook, Digitalization, Open Source, Ruby on Rails, Compound management

## Abstract

The development of an electronic lab notebook (ELN) for researchers working in the field of chemical sciences is presented. The web based application is available as an Open Source software that offers modern solutions for chemical researchers. The Chemotion ELN is equipped with the basic functionalities necessary for the acquisition and processing of chemical data, in particular the work with molecular structures and calculations based on molecular properties. The ELN supports planning, description, storage, and management for the routine work of organic chemists. It also provides tools for communicating and sharing the recorded research data among colleagues. Meeting the requirements of a state of the art research infrastructure, the ELN allows the search for molecules and reactions not only within the user’s data but also in conventional external sources as provided by SciFinder and PubChem. The presented development makes allowance for the growing dependency of scientific activity on the availability of digital information by providing Open Source instruments to record and reuse research data. The current version of the ELN has been using for over half of a year in our chemistry research group, serves as a common infrastructure for chemistry research and enables chemistry researchers to build their own databases of digital information as a prerequisite for the detailed, systematic investigation and evaluation of chemical reactions and mechanisms.

**Electronic supplementary material:**

The online version of this article (doi:10.1186/s13321-017-0240-0) contains supplementary material, which is available to authorized users.

## Background

In the field of organic chemistry, like in any research area, the availability of digital data is a prerequisite for a sustainable and successful research as it allows the access to results, the search for information, and the processing of the obtained research data [1–3]. Due to the ever-growing accumulation of information resulting from the constant saving and recording of data, it is imperative to improve data management with a digital system. Following the data life cycle, this enables the increase of knowledge by computing methods [4–6]. However, the lack of accessible and sufficiently mapped data limits the current research and the need to improve the situation was stated many times before [7–9]. Therefore, the maintenance of systems for digital data acquisition, management and storage is a key factor for an efficient research activity [10–12]. The need for digitalization of data and its systematic storage present challenges for the scientist, its institution providing the research infrastructure, and its scientific community. In the past, the discussion about the generation of and access to digital research information was mainly limited to published research data [10, 13, 14]. During the last two decades this accessibility has been improved drastically due to the availability of publications in online editions of scientific journals and the online-support of standard commercial databases like SciFinder [15] and Reaxys [16] as examples in chemical research. These developments have facilitated the search for the published information whereas solutions for a comprehensive digital storage and availability of all other research data, including data directly recorded in the laboratories, are still missing or lagging due to the challenging requirements of the research infrastructure in academia. The establishment of infrastructure in academic institutions is particularly difficult due to missing standards or policies in data handling and storage, very diverse work practices, also regarding to the used equipment, and the limited budget for fundamental improvements. In natural sciences, the digitalization of research data, as the basis for a later availability of the results and procedures, has to be implemented directly in the daily routine of the scientists. Specific aspects of the laboratory work have to be reflected in the electronic data acquisition and storage system depending on the research field. Although several electronic lab notebooks (ELNs) have been developed during the last years offering intelligent solutions for the documentation of research data (like SciNote [17], Biovia ELN [18], EMEN [19], Open BIS-ELN LIMS [20], LabFolder [21] and others [22–29]), only a very few electronic lab notebooks are dedicated to the chemical sciences [30, 31]. In chemical sciences in particular, challenges arise with the drawing and processing of chemical structures, a crucial and central step for the correlation of research data with the corresponding chemical transformation or structure [32]. Examples for systems in chemistry that offer the necessary support of chemical structures are the PerkinElmer E-Notebook for Chemistry [33], Indigo-ELN [34], LabTrove [35–37], and OpenEnventory [38]. These existing systems have already been in use by several groups and researchers. However, the sporadic implementation still reflects a mismatch between the offered solutions and the actual needs and resources of the chemists and their research facilities. This might be due to the high specific requirements for the software to reflect a fast moving research: suitable ELNs have to be readily obtainable, adaptable, and modulable without incurring additional costs. These features can probably only be offered by an Open Source project. In addition, a suitable, state of the art system for a sustainable research management should support the communication with additional external databases and repositories, as well as the connection to external devices and storage systems [39] of analytical results. Other important aspects are the embedding of calculation methods, and possible extension of the source code to the needs of other fields of chemistry (e.g. surface chemistry) and related domains of research (e.g. biology). As the identified criteria for a system to face the challenges of professional data management in academia could not be fulfilled by the currently available Open Source systems, we initiated the development of a powerful ELN for chemical sciences. Such an ELN should offer the features, currently lacking in available systems, while being flexible referring to the internal structure. Future extensions and adaptions to the needs of progressive chemistry research should be possible with minimal efforts. The development of the Chemotion ELN resulted in such a modern infrastructure that offers intelligent support of academic research projects being a key instrument for the acquisition, storage and management of digital data in chemistry.

## Implementation

**Fig. 1:**
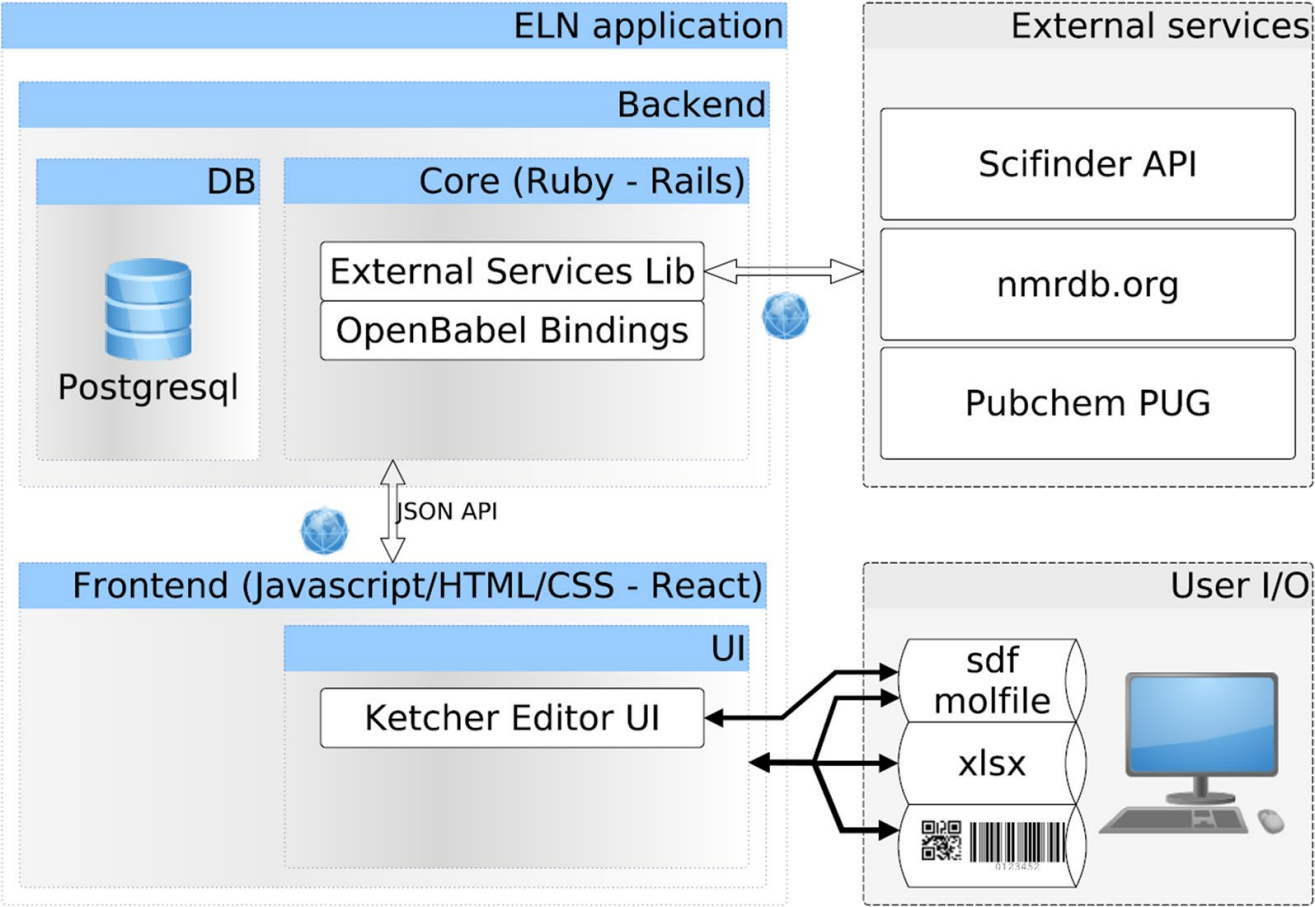
ELN architecture diagram: summary of programming languages, front-end input/output, and external service connection

The Chemotion ELN was programmed in Ruby, Javascript, HTML, and CSS. The backend server is built on the Ruby on Rails framework with PostgreSQL relational database, while the front-end user interface is mainly constructed with the ReactJS framework to serve a single page application (Fig. 1). Ruby on Rails adopts Ruby, a script language, which enables fast development with a clear model-view-controller (MVC) structure. On the other hand, ReactJS separates document object model (DOM) manipulations from data flow, decomposes entangled structures for sophisticated user interactions. People who want to expand features on the Chemotion ELN or start a new related project can comprehend the logics with a less steep learning curve. Ruby package management allows to easily implement external package from public code repository. The ELN was programmed in a way to be customizable through this practical package management. Plugins specific to the ELN can also be written as RAILS engine so to extend the ELN DB, server-side functions, but also the user interface. Adding additional web pages, or even modifying the main application page produced with ReactJS modules is possible.

## Results

**Fig. 2:**
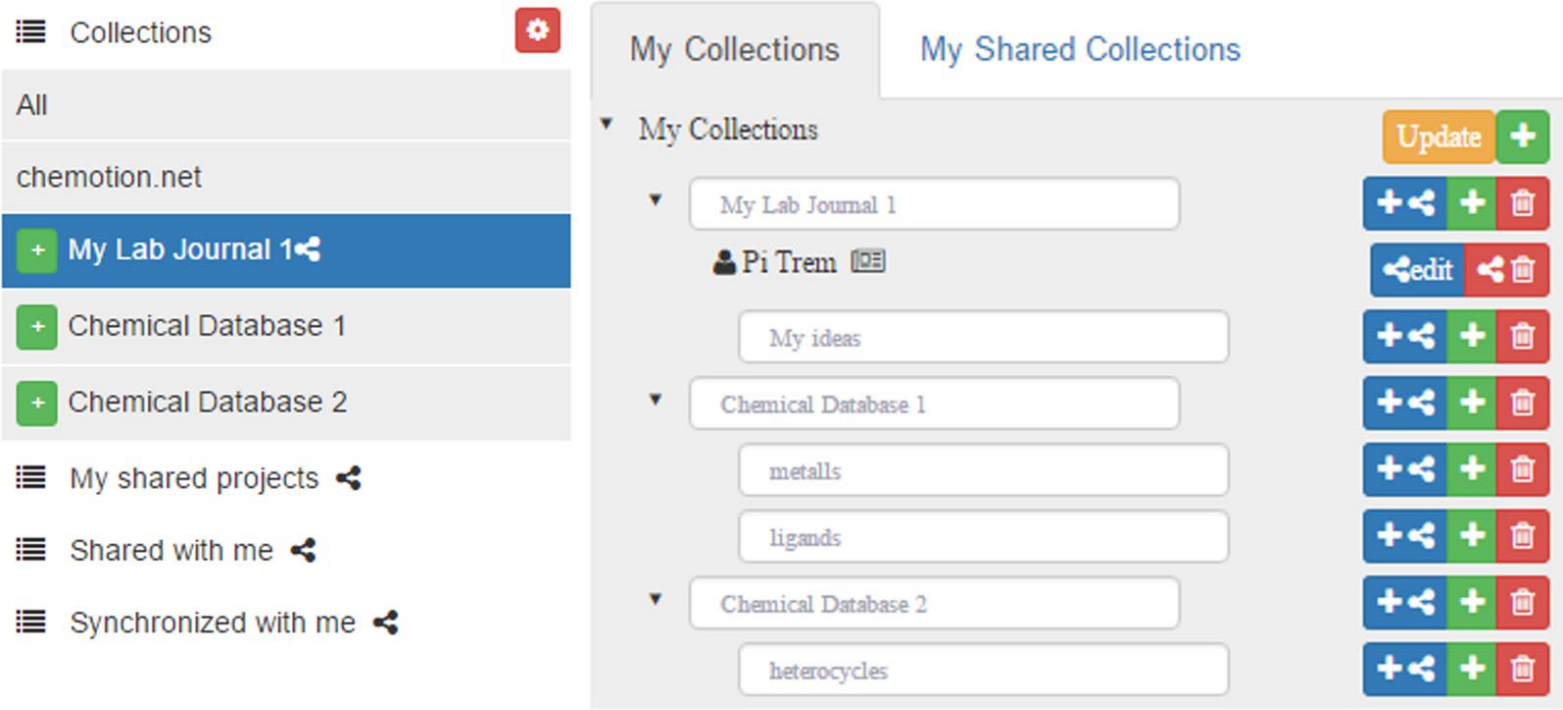
Management of collections as project planning and organization tool of the Chemotion-ELN: Left: management of projects and visibility of connections; Right: view within the ELN navigation bar

The Chemotion ELN offers an extended management system for projects allowing the formation of a clear structure for research data. The organization of projects is implemented by the sorting of individual elements according to *collections*. *Collections* can be generated, edited and deleted via a separated organizer which enables the establishment of a user defined ELN structure. Changes within the *collections* can be easily performed via drag and drop of selected elements allowing a fast hierarchal organization of collections of elements. This organization can be modified at any time, reflecting possible changes of the research projects in a flexible manner (Fig. 2). While the user management interface facilitates the work with information of the ELN user, it also contains management functionalities for the organization of information that has been gained from other researchers or that has been provided to other researchers.

### The core functions of Chemotion-ELN

**Fig. 3:**
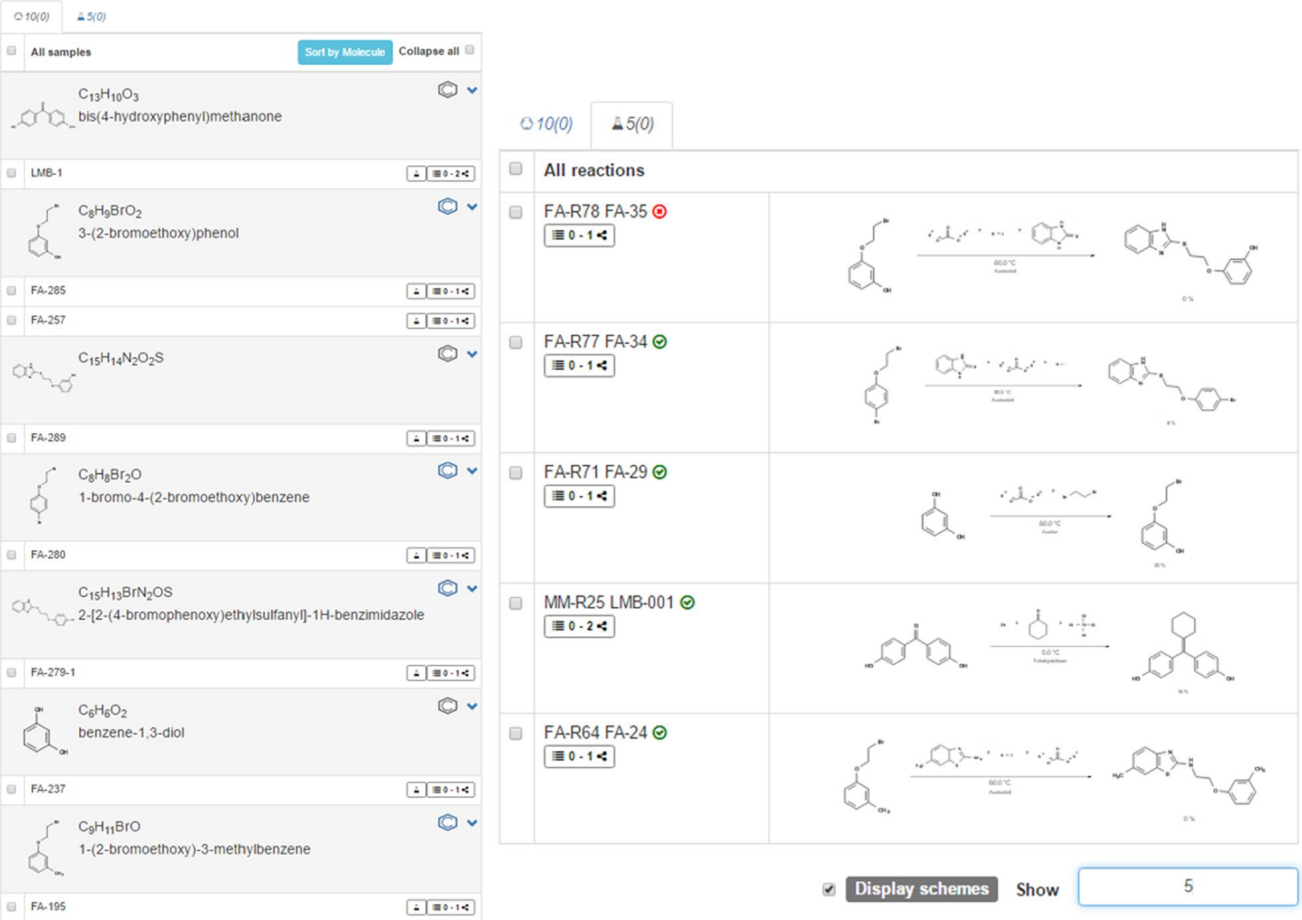
Organization of elements (molecules and reactions) in lists. Left: a selected list for molecules and samples with annotations for additional information. Right: a list of reactions with information on reagents, yield and additional notes

The ELN offers the necessary features for the documentation of chemical projects, including the processing of molecules and reactions. The elements of the ELN are organized in separate lists, e.g. for molecules or reactions, assigned to collections. This allows a clear and arranged structure at a low information level (Fig. 3). The list view is complemented by a summary of the available information on the single items such as the availability of data in external databases, the assignment to particular collections and the status of the stored attachments. Additionally, the list view supports a swift navigation to activities that are assigned to the list items. Another panel with a detailed level of information is visible upon selecting an element. This panel permits the user to visualize information and edit them. Textual descriptions, additional values, supplemental analytical data, links to external sources, and references are encompassed in several tabbed panels. The element lists and the detailed views of the selected elements are built with functionalities of a modern web-based application facilitating the fast organization of research data through diverse actions, such as drag and drop, automated sorting of elements, and notifications. The available information and the occurrence of the elements in other projects of the ELN are provided as a link.

### Elements of the ELN

**Fig. 4:**
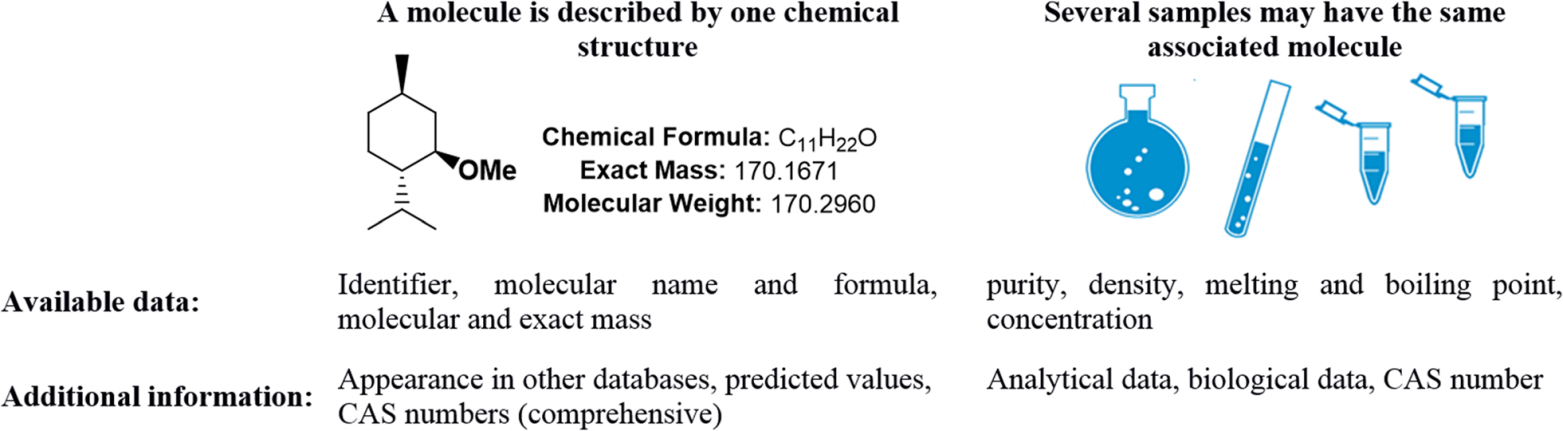
Differentiation between a molecule and corresponding samples

The submission of elements such as *molecules* and *reactions* is based on the use of an advanced embedded molecule editor derived from Ketcher, an Open Source web application [40]. The internal structure of the ELN follows strict rules for the creation of new elements which results in a differentiated database model having distinct tables for *molecules* and *samples* (see Fig. 4 and database relations in the Additional file 1). According to this concept, the generation of the molecular structure for a chemical compound requires at least the registration of a *molecule*. The structure editor is the essential part for the definition of molecules within the ELN as it generates the connection table. With this information, the International Chemical Identifier (InChI) and InChIKey, a hashed version of the InChI are generated by OpenBabel. With the database molecule table indexed over the InChIKey values, a new molecule entry is created if the unique identifier is not found. In that case, generic information is generated by OpenBabel and complemented by querying the PubChem database. This information comprises the molecule IUPAC name, the exact mass, the molecular mass, as well as SMILES code and the chemical abstracts service (CAS) registry number. The molecular structure of the *molecule* in combination with the assigned information then serves as a substantial part for the creation of *samples*, which are the physical equivalent to the designed molecules. Only samples can be assigned to research actions and reaction plans. The DB structure of the *sample* allows adding more information to a given theoretical molecular structure and includes the properties that depend on a specific experimental case such as the purity. The registration and consequent use of either *molecules* or *samples* while working with the Chemotion ELN is the basis for a well-organized and in the end, reproducible synthetic documentation. The association of samples to molecules allows the cumulation of information while offering flexibility in the definition of single samples and their visualization. As an example, MDL molfiles are stored both for the sample and its associated generic molecule giving the opportunity to individually style samples created from the same molecule. A very similar procedure is established for the assignment of CAS registry numbers of which all available ones are stored with the molecule allowing the user to select and store one of them with a particular sample (a detailed description of the process is given in the Additional file 1). While such a clear differentiation between molecules and samples is not reflected in most of the other chemistry ELNs, this is a central point in the development of the Chemotion ELN.

The definition of unique experimental samples in contrast to generic molecules is a prerequisite for a systematic documentation and follow-up of particular batches in the synthetic work process. Complemented with a naming of the individual sample that reflects the sample’s ancestry (the labels of descendent-samples include the label of the original sample and a systematic batch-number), the research workflow in the laboratory can be recorded with the highest accuracy.

The representation of a physically used substance or its preparation in the ELN includes the summary of the available data from the related molecule allowing a fast availability of all information that is necessary for a fast management of the research projects. The automatically provided data, as well as the input given by the user, are organized in three main tabbed panels which consist of

1. information for a detailed definition of the properties (Fig. 5, left),

**Fig. 5:**
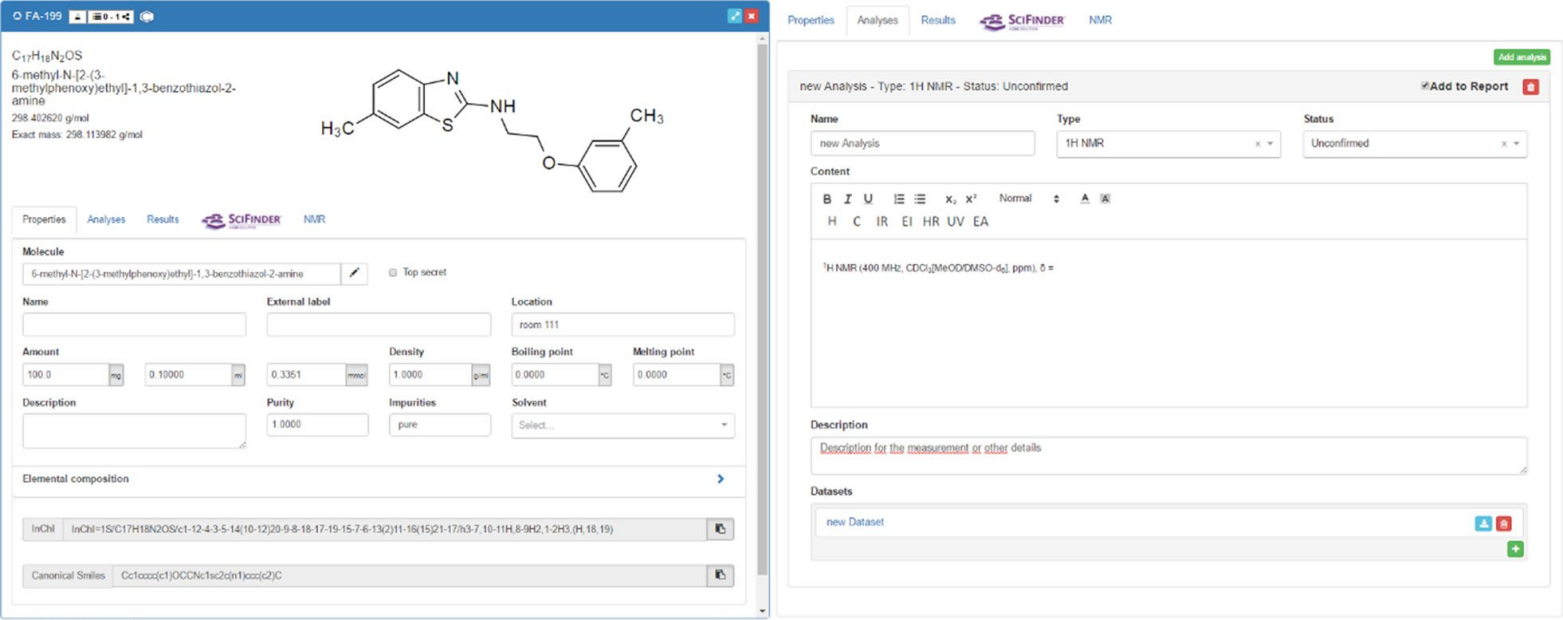
Left: detailed view of a properties tab including information of *molecule* and *sample* properties. Right: view of the analysis tab of the given *sample*
2. additional data that can be attached to the uploaded files with research data (Fig. 5, right),
3. results that have been gained with the sample through an external process.
  Other panels can be added through the ELN customization with plugins that provided the user extended functions:
4. request to SciFinder and a direct connection to the search results.
5. predicted NMR information via the web service NMRdb [41].

**Fig. 6:**
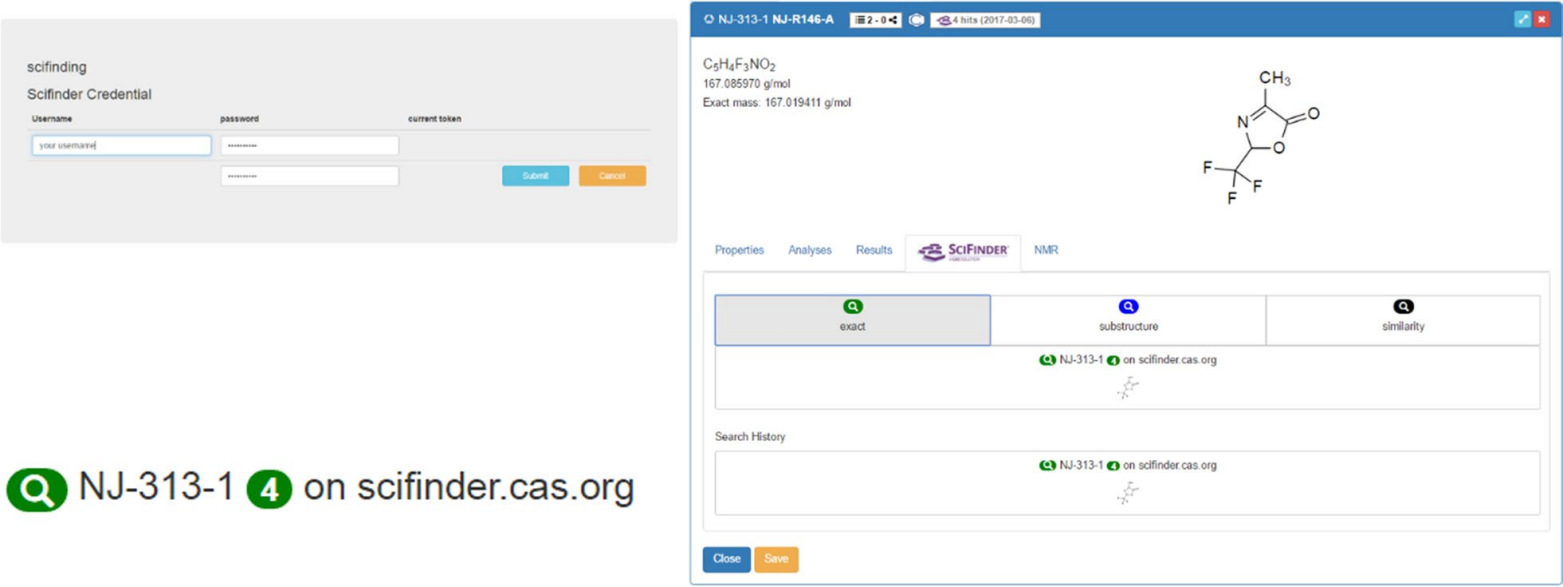
Left: changing the user settings with the SciFinder credentials to obtain a user token with time-limitation. Right: SciFinder tab with results of a database request with 4 hits identified for the exact structure search

The embedding of SciFinder functions (tab 4) requires the configuration of an ELN plugin which is also available on a public repository. However, the institution dependent credentials for the SciFinder service need to be configured on the server. The user access to SciFinder can be initialized via the change of the ELN-settings, where the CAS-provided credentials have to be entered once (Fig. 6). This step automatically generates an access token with a 10-day validity.

**Fig. 7:**
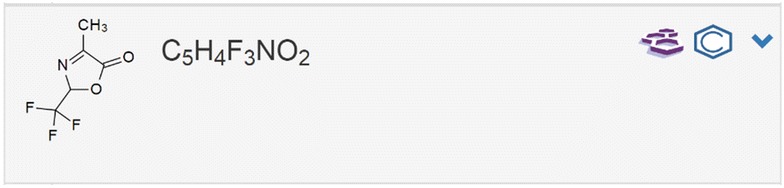
Request for presence and accessibility of information to specific molecules via PubChem and embedding of the answer sets in Chemotion ELN

The plugin implements query functions to the CAS SciFinder database according to three different search modes reflecting the SciFinder internal search modes “exact”, “substructure” and “similarity” search. The hit count of the search results is retrieved with a link to the answer set directing to the SciFinder web application. The history of the latest requests and answers of the current user is also listed. As soon as a molecule search in SciFinder is processed, the results are also given in the list of molecules, indicating the search date, whether the structure is registered in SciFinder or not and the number of results. The direct visibility of published structures via the ELN allows a fast access to information which was, up to know, only to be retrieved via the SciFinder page directly. To give a comprehensive overview of the novelty of a researcher’s work and the availability of research data, we additionally implemented an automated procedure to assess the presence of any molecule from the ELN in the PubChem database (NCBI). As given for the embedded SciFinder feature, the matching molecules are accessible via a direct link to the PubChem Index of the identified item. The information on the presence of the requested structure in the NCBI database is summarized in the molecule and sample lists (Fig. 7). While the SciFinder search allows a differentiation of the search request according to the user’s preference, the implemented PubChem requests are only executed with the exact structure. While being less flexible according to customized search strategies, this limitation allows the automated processing of the requests instantly with the creation of a new molecular structure.

**Fig. 8:**
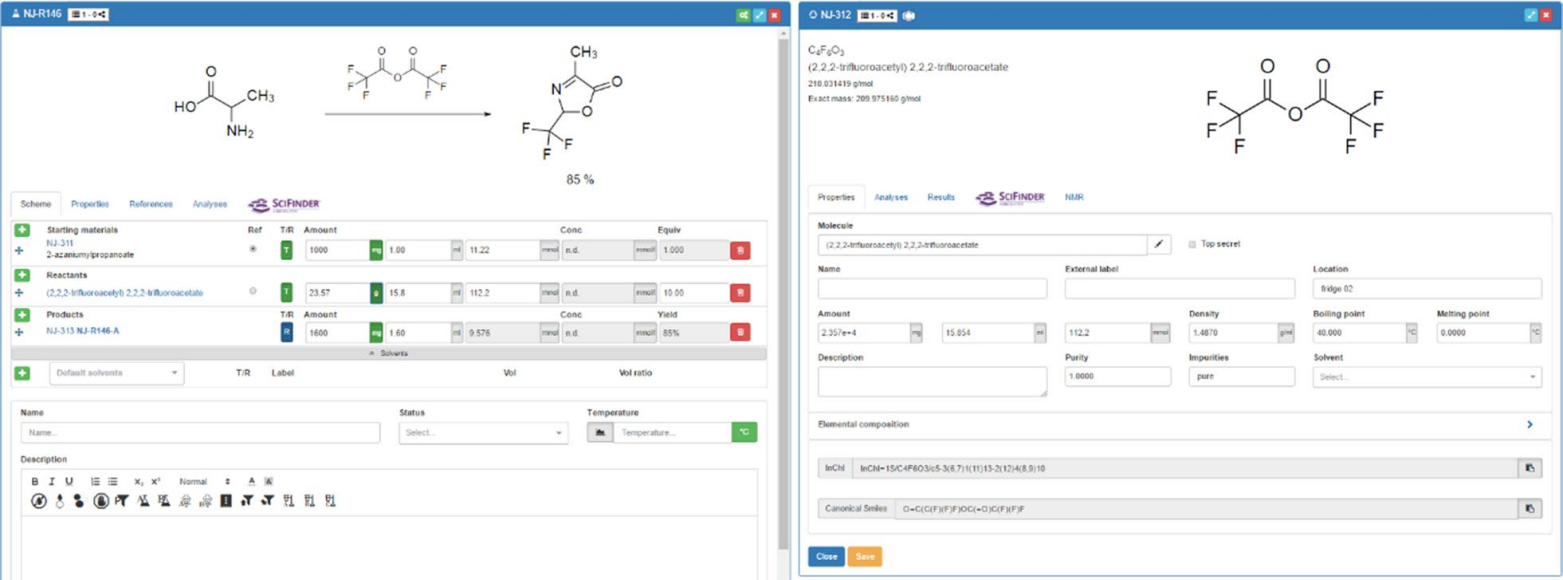
Planning and editing reactions via the Chemotion ELN (left panel): direct connectivity to the sample related data (right panel)

Besides *molecules* and *samples*, *reactions* belong to the main elements that can be generated and managed with the ELN. A reaction is created easily by the addition of information to a reaction template (Fig. 8). The user can assign samples and molecules to the reaction in their distinct function as starting material, reagent or product. The basic scheme for samples in reactions allows the addition of the amount of the substances in g (alternatively in mg or µg), in ml (or µl) or the definition of the used compound in mol (mmol) equivalents. The implemented dependencies between the given information and the molecular weights allow the calculation of all necessary values as long as the basic information is given. The structure of the reaction user interface is very flexible enabling the exchange of elements at any time per drag and drop. Samples that have been assigned to a role as starting material can be changed into reagents during the planning of the reaction. The assignment of samples to particular roles within a reaction act upon the calculations, as the equivalents are always calculated with respect to the given amount of starting material which is set to 1 per default. When several starting materials are entered, either one of them, or a reactant, has to be set by the user as the reference material with 1.0 equivalent. A unique feature of the Chemotion ELN is the record of real values in parallel to the data of the originally planned experiments. This allows the accurate documentation of the real experiment while having the possibility to use the planned procedure as a template that can serve as a copy for a repeat in a standard way. The change from target to real values is implemented via a switch from value *T* to *R* for each sample. The chemicals that are assigned to the reaction are accessible via a direct link to the detailed level of the sample list. All data and changes that are submitted to the samples (like the density of a chemical) are considered instantly for the calculation of the reaction. The ELN is designed on the one hand to offer as much flexibility as possible but on the other hand to limit user actions that could compromise the integrity of the experimental data. While all parameters of a reaction can be inputted and submitted either via the predefined or free text fields within the information panels like under the *Scheme* tab, there are other fields where calculated data are only visible but not editable. An example for the latter limitation is the yield field displayed for reactions. The ability of inputting a value for the yield of a reaction is disabled in all cases, as the yield should be the result of the gained amount of the product of a reaction. Another feature for the planning of reproducible reactions has been added with a solvent manager. This tool allows the addition of several solvents (via drag and drop from the sample list, via drawing and generating a solvent from scratch, or via a selection from a dropdown menu) and volumes, for which the concentration of reagents is estimated automatically and given in the reaction table (Fig. 8).

The Chemotion ELN can be used for a detailed tracking of samples and reactions thanks to a systematic and automatic identification of all items, including an intuitive labeling of the given workflow. Samples that are part of any process within the ELN bear information about their origin and use in their name and short_label descriptors. Samples that have been newly created or that have been generated via the copy of a molecular structure have a simple name consisting of the initials of the ELN user and a sequential number. Samples that are created from those samples are regarded as child-samples which is visible through the attachment of a child batch number “− 1..− x” to the original label. Samples that appear as a target compound in a reaction gain in addition a reaction label which allows the direct assignment of this sample to the reaction and its number. Therefore, the systematic reaction name appears in every product, side product and fraction of the experiment allowing for a fast identification of analytical results being labeled in the same manner. All samples that are assigned to the type starting material or product are visible via the sample and molecule list, while samples that are assigned to the function reagent are not listed. This allows a brief representation of the important information by avoiding overcrowding the interface with repeatedly used standard reagents (e.g. inorganic salts, bases) and by keeping a consistent record of all reagents used. The reaction scheme and the reaction table can be completed by additional information such as *name* [free text], s*tatus* [planned, successful, unsuccessful], *temperature* or time–temperature table [number/adaptable to °C, °F, K], and *description* (free text). The addition of a description is supported by several predefined and formatted procedures which might be used for a fast report on a chemical procedure in a standardized manner. Three other tabbed panels have been implemented for the submission of further information to a reaction: under tab *properties,* the start and end time points of a reaction and the detailed definition of the TLC control can be given. Literature citations can be added to the reaction by typing a *title* and the corresponding *URL* in the *references* tab, which allows the addition of as many references as desired. The last tab, *analysis,* displays the analytical experiments associated to each of the obtained product samples of the reaction. This allows a clear and straightforward organization of the obtained analytical results even if several isolated compounds have been obtained. The user benefits from several direct export functionalities working with reactions in the detail level: the information that is distributed over the described four tabbed panels can be summarized either in one word document in a very practical manner or the samples that are used in the reaction can be exported to Excel with one mouse click.

### Export and import

**Fig. 9:**
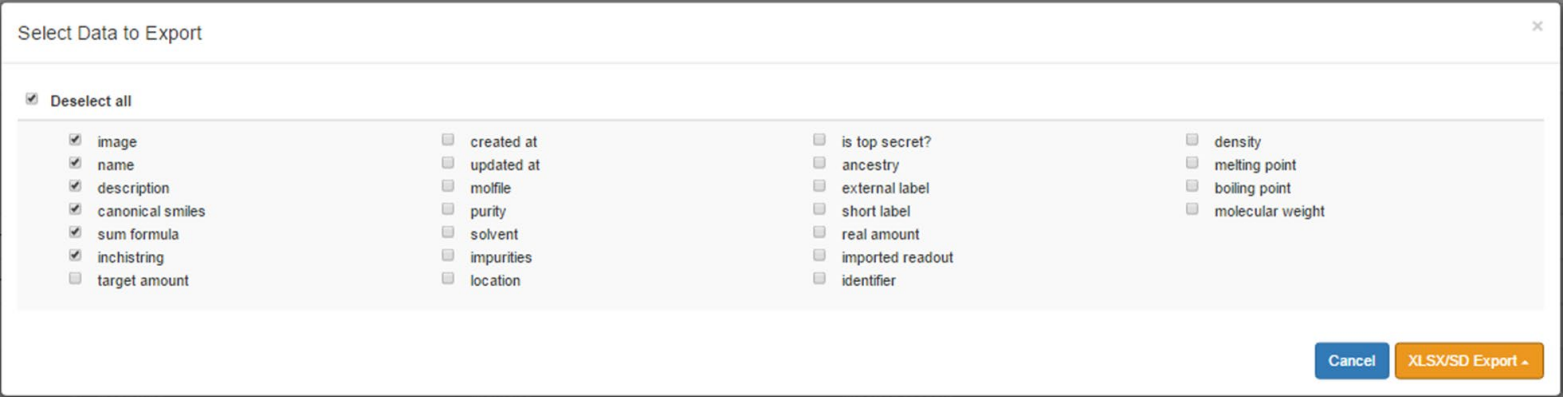
Export scheme allowing the selection of single items to be exported

Exchanging data between different or isolated systems is a critical issue while managing data. For this reason, the support for two simple and widely used file formats has been implemented and allows transferring data for a selection of samples in and out of the ELN as Excel (.xlsx) or sd files (.sdf). The details level of data to export can be determined by the user via a check box menu (Fig. 9).

### Sharing of information

**Fig. 10:**
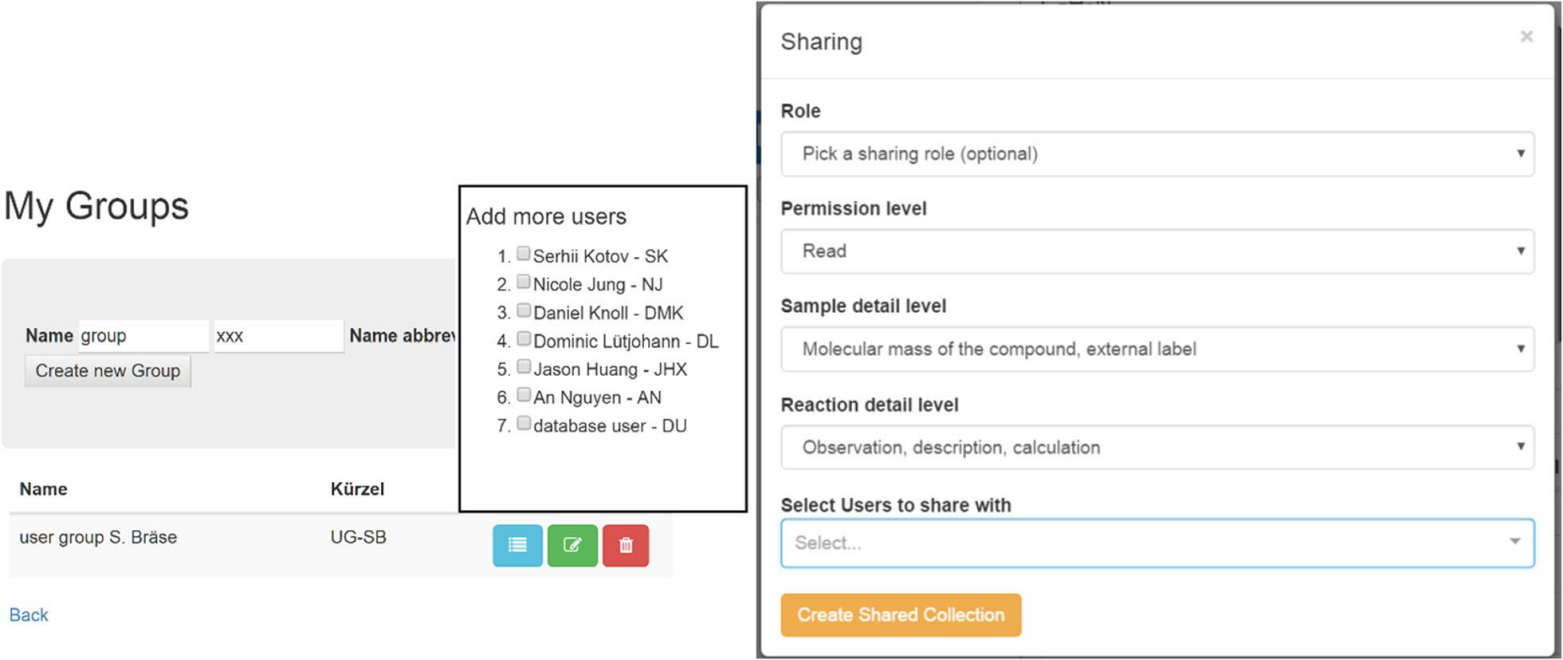
Left: creation of new user groups; Right: definition of user groups and assignment of sharing role, permission level and available detail level for single users and user groups

The Chemotion ELN was equipped with two functions of sharing information with other ELN users. These tools complete the functionality of exporting and importing information allowing the detailed visibility of the obtained research data directly through the ELN. Both operation models, called sharing and synchronization, are accessible through a user interface that allows the organization of single colleagues or groups according to their status and desired access policies (Fig. 10, right). The ELN user and owner of the submitted data sets the level of permission for the recipient, or group, either by choosing a standard role or by selecting more detailed information levels. The permission levels for allowed actions range from a simple *read* policy to a *take ownership* policy. The detailed level of what data can be accessed for the samples and reactions can also be limited to a few fields. User groups are easily defined to facilitate the sharing of the research activity with a larger community (Fig. 10, left).

Though the selection of the user role and rights are the same for the sharing and synchronizing tool, the two options are different concerning the currentness of the provided research data. Through the ‘sharing’ of a collection, a fixed set of samples and reactions is made accessible to others with, if desired, the ability for the recipients to edit the contained elements. The actions *read*, *write*, *share*, *delete*, *import elements* or *take ownership* depending on the access policy can be used, but new elements cannot be added. This is however feasible when using ‘synchronized’ collections. Synchronized collections are created to allow a permanent access of other ELN users to the chosen set of research data including the visibility (and modification) of changes that have been made after the synchronization.

### Search functions

**Fig. 11:**
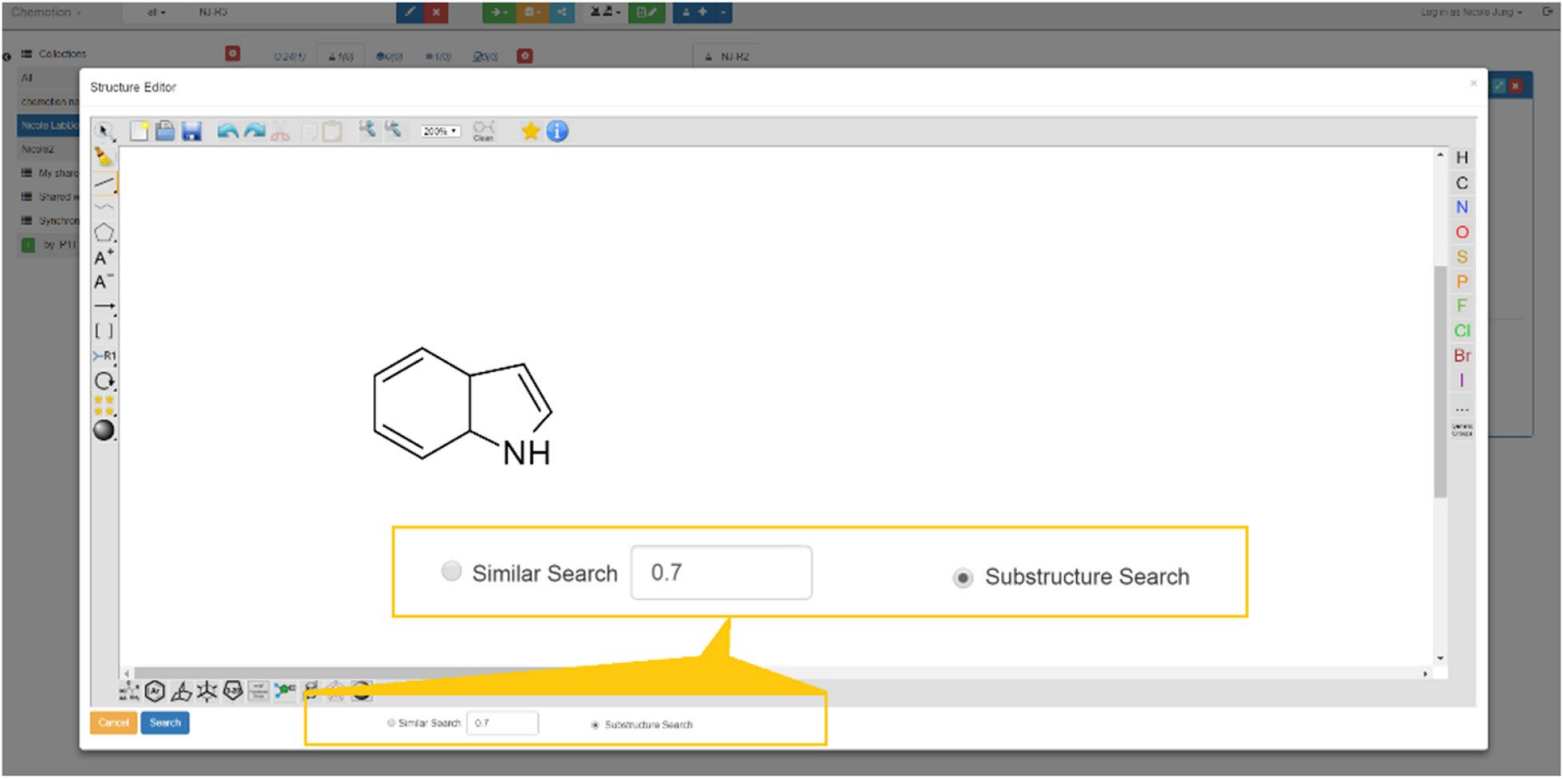
Search functions with structure and substructure search (adaptable through similarity search)

One of the main arguments for the management of research data with an ELN is the digital availability of information. The digital availability offers the possibility to search for data and information if the organization and maintenance of the ELN supports that in a suitable way. The Chemotion ELN allows text and structure search within diverse contents of the ELN. The search of either text fragments or chemical structures can be further limited to distinct elements (samples, reactions) to facilitate the evaluation of the results. The text based search uses the postgresql trigram module for alphanumeric trigram matching to seek the presence of text or formula fragments in samples. Most of the non-numeric properties of the samples such as: *name*, *molecule formula*, *IUPAC name*, *inchistring* and *canonical smiles* are searched. The associated content in reactions will be filtered based on the search result. The search for structures can be performed either by the search for a substructure or a similarity search of which both methods are fingerprint-based methods. We implemented a path-based fingerprint method, referred as FP2 in OpenBabel. This fingerprint is identical with Daylight fingerprints, which are used as a standard for benchmarking in many publications and is also used to calculate molecule similarity using the Tanimoto coefficient [42]. The minimum similarity threshold can be defined through the ELN interfaces (Fig. 11).

### Codes and tracking

**Fig. 12:**
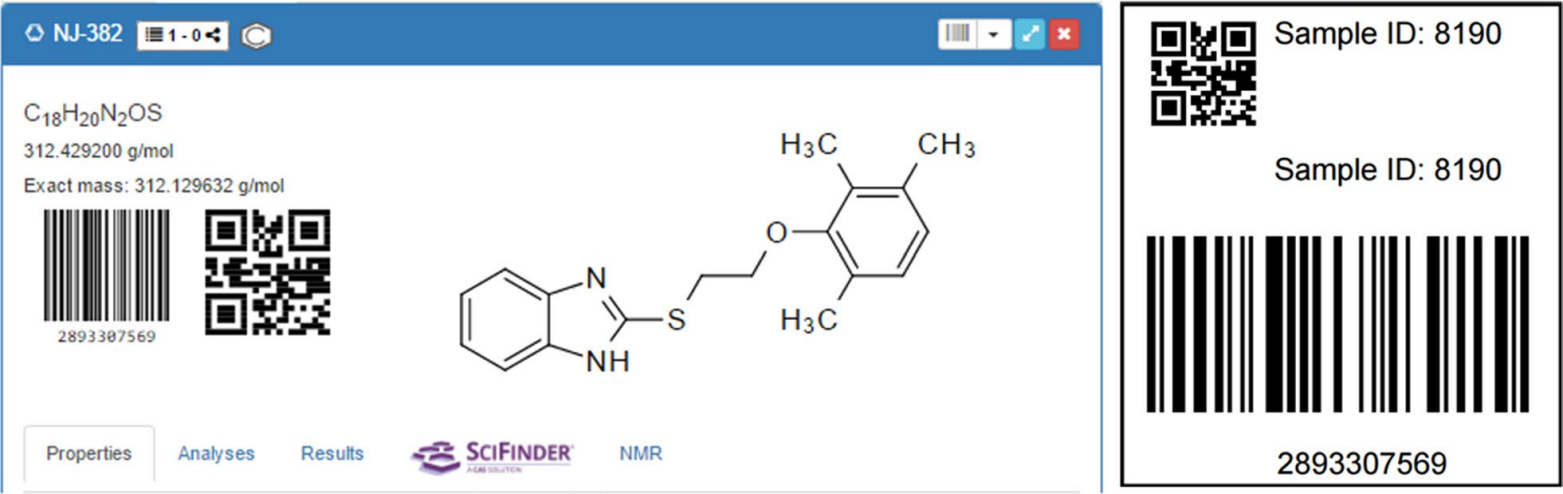
Barcode and QR code generation, printing and tracking of samples via code reading

The management options of the Chemotion ELN are complemented by a barcode and QR code tracking of single elements and items. This feature, often offered with laboratory and information management systems (LIMS), is implemented for reactions, samples and analyses. Parallel to the creation of each of the latter items, a Universally Unique Identifier (UUID) version 4 is registered. The ELN provides a QR code or a truncated barcode representation of the associated identifier allowing a flexible labeling. Analyses associated to samples are also assigned to a UUID. Procedures to generate pdf files of the codes for a fast printing in different sizes have been implemented, and render the QR code, the Barcode and the assigned Sample ID (Fig. 12). Using a webcam or a specific code reading device, the user can scan the code and navigate directly to the associated element in the ELN.

### Evaluation of the ELN and user feedback

The development of the Chemotion-ELN is a result of long lasting process within our work group aiming for the installation of software that fulfills the requirements of a modern, fast and flexible infrastructure. The ELN is used in our group by master students, PhD students and technicians. The continuous integration and deployment provide the users the latest developments, changes, and corrections on a frequent basis (at least once a week). In this manner, the ELN is constantly checked and evaluated allowing the fast identification of errors and missing features. New feature requests or suggestions are entered by selected users via an internal GitLab CE portal and are prioritized according to urgency and users’ upvoting. The user’s feedback reveals roughly two groups: users who have tested or used other ELNs before and those who use an ELN for the first time.

For the first user group, the feedback is consistently positive and the training time to an experienced user is short. This group has remarked the fast and convenient way to search items (samples/reactions) and the clear overview of all data that can be adapted to the user’s preferences. Users of this group extensively use features for storing NMR spectra along with the experiments and for sharing results, reactions, as well as whole collections of entries with colleagues. When asked about the main differences compared to other systems, they emphasize a better and more sustainable accessibility to their data because the use of the ELN is not limited to the availability of particular addition software and can be accessed independently of the platform. While with former ELNs, the risk to not access the data any more as a result of software or hardware problems, was discussed very often, the Chemotion-ELN was very successful in providing confidence in the accessibility of digital data. Especially the latter argument is interesting because it stands in contrast to the opinion of the non-ELN-experienced user group. These users fear, which is one of their strongest arguments against a use of the ELN, that the system could be compromised from outside and that research data could be stolen or deleted.

The non-experienced ELN users need more time to become familiar with digital reporting in general, as they e.g. need to understand the logic of e.g. the differentiation between molecules and samples and its use within the ELN. For these users, teaching or mentoring by more experienced users is very important to become familiar with all functions. We tried to advocate the use and functions of the software to the students with a manual that includes illustrative examples and screenshots of all features. It turned out that such a written manual has little impact to raise the user interest. Functionalities that are valued by all users are for example the SciFinder function and moreover the PubChem link as well as the retrieval of CAS registry numbers. Those functions allow a fast retrieval of additional information on compounds or possible reactions or properties and are therefore highly requested. The individual use of the provided ELN depends strongly on the preferences of the users and on the equipment of the laboratory in general. The majority of users appreciate the availability of their data wherever they are. Although this depends of course on the accessibility to internet, and a VPN connection. It allows them to be more flexible in their time management because reviewing of data, collecting of information and additional documentation from different workplaces can be done at any time. As all PhDs, master students, and technicians spend most of their working time in the laboratory, the main application of the ELN takes place directly in the chemistry lab and all users enter the ELN either via a personal notebook or desktop PCs that is provided. None of the current researchers uses the ELN via tablet or a smartphone (although there are no technical limitations). This is due to the fact that an important advantage of the ELN is the direct and connected visibility of datasets and information. This visibility is lost in parts with smaller screens. The ELN users are often asked about the need to further write paper-based notes and descriptions. At this stage, the ELN does not include the connectivity to devices so therefore everyone still needs to do hand-written documentation to some extend, at least to record information from external instruments like balances.

## Conclusion

We present the development of an Open Source electronic lab notebook (ELN) for researchers who work in the field of chemical sciences making allowance for the growing dependency of scientific activity on the availability of digital information. The web based application which has already been implemented in daily laboratory work allows the acquisition, management, storage, processing, and sharing of chemistry research data. The ELN as an example for a modern and powerful research infrastructure provides tools for communicating and sharing the recorded data. It facilitates research via offering the access to various functions, helper tools and external sources. In addition, it will allow one of the most important improvements regarding to the scientific work: it will enable chemistry researchers in academia to build their own databases of digital information which is a prerequisite for the detailed, systematic investigation and evaluation of chemical reactions and mechanisms. However, many features that are necessary to meet all needs for chemistry research, are not implemented yet and will be part of further developments. Examples for those work-in-progress features are (a) a document generation function that creates and archives projects as either a report or the supporting information for a publication, (b) the implementation of queries to additional chemistry databases like ChemSpider, and (c) the development of an API to a chemistry repository that will allow the direct transfer of research data to an online portal with global access. The developments are still ongoing and novel ideas for additional features are discussed daily with the programmers for future implementations. On a broader scope, additional functionalities have been requested by researchers working in the field of biology but cooperating closely with chemists. In future, the ELN should be useable as a platform that allows the sharing of information on molecules for the research on a common project. Although there is a need for adaptions and extensions of the current software version to meet those requirements, first results show already a good applicability of the ELN in an interdisciplinary work environment.

## Additional file

**Additional file 1.** Supporting information including (1) technical aspects and details covering the software development and the Docker file, (2) database entity-relationship diagram, (3) summary of features according to the main modules, and (4) procedures in pictures.

## Abbreviations

ELN: electronic laboratory notebook
MVC: model-view-controller
DOM: document object model
CSS: cascading style sheets
DB: database
NCBI: National Center for Biotechnology Information (US)
UUID: Universally Unique Identifier
VPN: virtual private network

## References

[1] Winkler-Nees S, Neuroth H, Strathmann S, Oßwald A, Ludwig J (2013). Status of discussion and current activities: national developments. Digital curation of research, experiences of a baseline study in Germany.

[2] Stajich J, Lapp H (2006). Open source tools and toolkits for bioinformatics: significance, and where are we?. Brief Bioinf.

[3] Owens B (2016). Data sharing: access all areas. Nature.

[4] Pirhadi S, Sunseri J, Koes DR (2016). Open source molecular modeling. J Mol Graph Model.

[5] Segler MH, Waller MP (2017). Neural-symbolic machine learning for retrosynthesis and reaction prediction. Chem Eur J.

[6] Christ C, Zentgraf M, Kriegl J (2012). Mining electronic laboratory notebooks: analysis, retrosynthesis, and reaction based enumeration. J Chem Inf Model.

[7] Campbell P (2009). Data’s shameful neglect. Nature.

[8] Bird C, Frey J (2013). Chemical information matters: an e-research perspective on information and data sharing in the chemical sciences. Chem Soc Rev.

[9] Alsheikh-Ali A, Qureshi W, Al-Mallah M, Ioannidis J (2011). Public availability of published research data in high-impact journals. Plos ONE.

[10] Szymkuć S, Gajewska EP, Klucznik T, Molga K, Dittwald P, Startek M, Bajczyk M, Grzybowski BA (2016). Computer-assisted synthetic planning: the end of the beginning. Angew Chem Int Ed.

[11] Borgman C (2012). The conundrum of sharing research data. J Am Soc Inf Sci Technol.

[12] Ghosh S, Matsuoka Y, Asai Y, Hsin K, Kitano H (2011). Software for systems biology: from tools to integrated platforms. Nat Rev Genet.

[13] Butler D (2017). Gates Foundation announces open-access publishing venture. Nature.

[14] Lawrence K (2017). Open access is evolving and ChemistryOpen is too!. Chemistryopen.

[15] Scifinder (2017) Chemical abstracts service. http://www.cas.org/products/scifinder

[16] Reaxys (2017) Elsevier. https://www.elsevier.com/solutions/reaxys

[17] sciNote, 1.9.0 (2017) BioSistemika USA. https://github.com/biosistemika/scinote-web

[18] biova (2017) http://accelrys.com/products/unified-lab-management/biovia-electronic-lab-notebooks

[19] Rees I, Langley E, Chiu W, Ludtke S (2013). EMEN2: an object oriented database and electronic lab notebook. Microsc Microanal.

[20] Barillari C, Ottoz D, Fuentes-Serna J, Ramakrishnan C, Rinn B, Rudolf F (2016). openBIS ELN-LIMS: an open-source database for academic laboratories. Bioinformatics.

[21] Labfolder. https://www.labfolder.com

[22] Rubacha M, Rattan A, Hosselet S (2011). A review of electronic laboratory notebooks available in the market today. Jala.

[23] Zeng J, Hillman M, Arnold M (2011). Impact of the implementation of a well-designed electronic laboratory notebook on bioanalytical laboratory function. Bioanalysis.

[24] Beato B, Pisek A, White J, Grever T, Engel B, Pugh M, Schneider M, Carel B, Branstrator L, Shoup R (2011). Going paperless: implementing an electronic laboratory notebook in a bioanalytical laboratory. Bioanalysis.

[25] Taylor KT (2006). The status of electronic laboratory notebooks for chemistry and biology. Curr Opin Drug Discov Dev.

[26] van Eikeren P (2004). Intelligent electronic laboratory notebooks for accelerated organic process R&D. Org Process Res Dev.

[27] Achour Z, Laidboeur T, Gien O, Musolino A, Bon X, Grimaud B (2004). Sanofi-synthelabo chemical development and the development of an electronic laboratory notebook. Org Process Res Dev.

[28] Walsh E, Cho I (2013). Using Evernote as an electronic lab notebook in a translational science laboratory. J Lab Autom.

[29] Goddard NH, Macneil R, Ritchie J (2009). eCAT: online electronic lab notebook for scientific research. Autom Exp.

[30] Bird C, Willoughby C, Frey J (2013). Laboratory notebooks in the digital era: the role of ELNs in record keeping for chemistry and other sciences. Chem Soc Rev.

[31] Voegele C, Bouchereau B, Robinot N, McKay J, Damiecki P, Alteyrac L (2013). A universal open-source electronic laboratory notebook. Bioinformatics.

[32] Coles S, Frey J, Bird C, Whitby R, Day A (2013). First steps towards semantic descriptions of electronic laboratory notebook records. J Cheminform.

[33] E-Notebook for chemistry. https://www.cambridgesoft.com/Ensemble_for_Chemistry/ENotebookforChemistry/

[34] Indigo, gga software. https://github.com/ggasoftware/indigo

[35] Day A, Coles S, Bird C, Frey J, Whitby R, Tkachenko V, Williams A (2015). ChemTrove: enabling a generic ELN to support chemistry through the use of transferable plug-ins and online data sources. J Chem Inf Model.

[36] Frey J, Coles S, Milsted A, Willoughby C, Bird C (2014) Sample management with the LabTrove ELN. From abstracts of papers, 247th ACS national meeting & exposition, Dallas, TX, United States, March 16–20, 2014, CINF-44

[37] Willoughby C, Bird C, Coles S, Frey J (2014). Creating context for the experiment record. user-defined metadata: investigations into metadata usage in the LabTrove ELN. J Chem Inf Model.

[38] Rudolphi F, Goossen L (2012). Electronic laboratory notebook: the academic point of view. J Chem Inf Model.

[39] Lütjohann D, Jung N, Bräse S (2015). Open source life science automation: design of experiments and data acquisition via “dial-a-device”. Chemom Intell Lab Syst.

[40] Ketcher, gga software. https://github.com/ggasoftware/ketcher

[41] Banfi D, Patiny L (2008). www.nmrdb.org: resurrecting and processing NMR spectra on-line. Chimia.

[42] Bajusz D, Racz A, Heberger K (2015). Why is Tanimoto index an appropriate choice for fingerprint-based similarity calculations?. J Cheminform.

