# Supplementary material for: Chemotion ELN: an Open Source electronic lab notebook for chemists in academia
Source: J Cheminform. 2017 Sep 25;9:54. doi: 10.1186/s13321-017-0240-0 (PMC5612905; doi:10.1186/s13321-017-0240-0)
Supplement: Supplementary file 1 — Additional file 1. Supporting information including (1) technical aspects and details covering the software development and the Docker file, (2) database entity-relationship diagram, (3) summary of features according to the main modules, and (4) procedures in pictures. [file 13321_2017_240_MOESM1_ESM.pdf]

Chemotion ELN: An Open Source Electronic Lab Notebook for chemists in academia

Pierre Tremouilhac[a], An Nguyen[a], Yu-Chieh Huang[a], Serhii Kotov[a], Dominic Lütjohann[a§], Florian Hübsch[c], Nicole Jung[a,b], Stefan Bräse*[a,b]*

[a]Institute of Toxicology and Genetics, Karlsruhe Institute of Technology, Hermann-von-Helmholtz-Platz 1, 76344 Eggenstein-Leopoldshafen, Germany; [b]Institute of Organic Chemistry, Karlsruhe Institute of Technology, Fritz-Haber-Weg 6, 76131 Karlsruhe, Germany; [c]Ninja-Concept GmbH, Haid-und-Neu-Straße 18, 76131 Karlsruhe, Germany.

Contents

1. Technical Aspects and Details covering the Software Development and the Docker File 3
   1.1 Technical aspects and programming details 3
   1.2 Docker File........ 3
2. Database Entity-Relationship Diagram........ 5
3. Summary of features according to the main modules........ 6
4. Procedures in pictures 7
   4.1 Research management in collections 7
   4.2 Organization of single elements and listing 9
   4.3. Detail level for samples........ 11
   4.4 SciFinder Credential and requests........ 15
   4.5 Listing of database requests 15
   4.6. Planning and editing reactions 16
   4.7 Configuration of export functions and results (examples) 18
   4.8 Sharing of information (single user and group level) 19
   4.9. Search functions 21
   4.10. Coding and Tracking 22

1. Technical Aspects and Details covering the Software Development and the Docker File

1.1 Technical aspects and programming details

The Chemotion ELN was programmed in Ruby, Javascript, HTML, and CSS. The backend server is built on the Ruby on Rails framework with PostgreSQL relational database, while the front-end user interface is mainly constructed with the ReactJS framework to serve a single page application. Ruby on Rails adopts Ruby, a script language, which enables fast development with a clear MVC (model-view-controller) structure. On the other hand, ReactJS separates DOM (document object model) manipulations from data flow, decomposes entangled structures for sophisticated user interactions. People who want to expand features on the Chemotion ELN or start a new related project can comprehend the logics with a less steep learning curve. Ruby package management allow to easily implement external package from public code repository. The ELN was programmed in a way to be customizable through this practical package management. Plugins specific to the ELN can also be written as RAILS engine so to extend the ELN DB, server-side functions, but also the user interface. Adding additional web pages, or even modifying the main application page produced with ReactJS modules is possible. The extension with SciFinder or NMRdb functions are two already mentioned examples.

1.2 Docker File

Setting up the development environment can be a tedious task for the software engineer. The ELN project is programmed by Ruby-on-Rails as the web application, connecting to the PostgreSQL, and npm for client side package manager. On the back-end we are using OpenBabel as chemistry helper, Rmagick for the Ketcher editor and many more packages. On the front-end, browserify is used to convert Nodejs packages for use in the browser. Getting everything ready is a time-consuming task for people wantingtodeploy or develop our ELN project. For the ease of our open-source development, we also provide a Docker configuration for our ELN project using Docker.

Docker[1] is an open-source project that helps simplify process building, shipping, and running web applications. Docker wraps the project code with all the needed information and dependencies for the application into one unit called a "container". There are two ways for users or developer to setup the development environment:

[1] https://www.docker.com/

- Get the *Dockerfile*[2] from our repository[3]. *Dockerfile* is a script for Docker to install every software package that is required by the ELN: the PostgreSQL database, the back-end Ruby-on-Rails with additional Ruby gems (using Ruby bunder[4]) to front-end npm[5] package.
- Directly pull the docker image from Docker hub[6]. This image actually is the compressed docker image from the *Dockerfile*.

After creating or downloading the Docker image, the ELN project is ready to use by starting a Docker container from the Docker image.

---

[2] https://docs.docker.com/engine/reference/builder/
[3] https://github.com/ComPlat/chemotion_ELN
[4] http://bundler.io/
[5] https://www.npmjs.com/
[6] https://hub.docker.com/u/complat/

2. Database Entity-Relationship Diagram

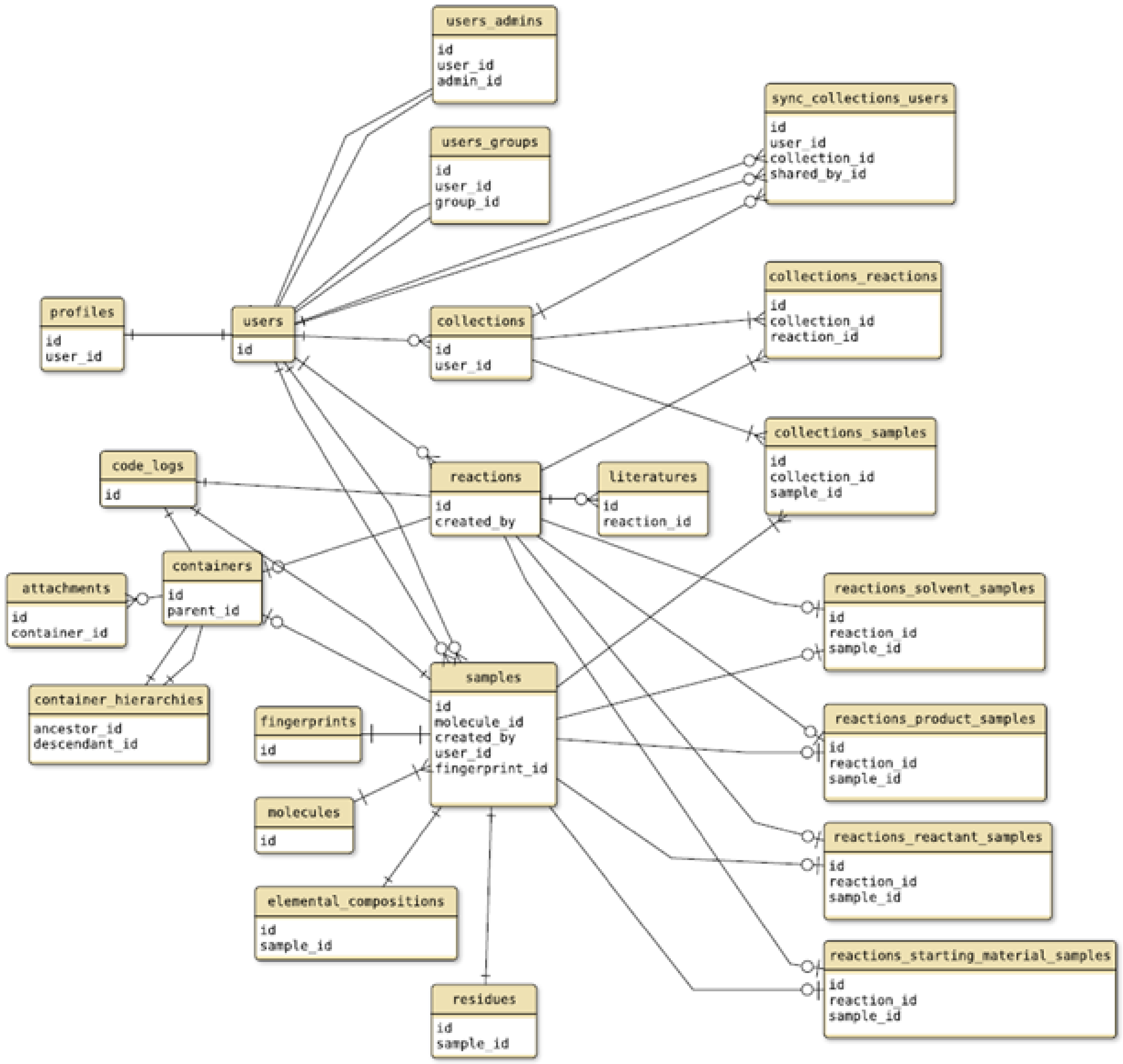

3. Summary of features according to the main modules

Field	module	Features
ELN-Structure	Management of collections	• Management of standard collections<br>• Management of shared collections
	Organization in Lists	• Automatic sorting according to molecule and latest updated item<br>• Sorting of all samples to corresponding molecular structure<br>• Availability of literature citations<br>• Availability of analysis and collection information
	Detail level view	• Organization in tabs<br>• Visibility of several detail windows in parallel
	Information Tabs	• Support of text-fields and drop-down menus<br>• Availability of external databases (direct visibility and link)
	Data and attachments	• Attachment of research data in diverse file formats
Elements	Samples	• Manifold input fields for a detailed description<br>• Consistent, automatic, labeling of samples and their derived items<br>• File format: Molfiles, InChI and Smiles<br>• CAS-number (*via* PubChem)
	Reactions	• Consisting of samples and their child-samples<br>• Automatic retrieval of information and values from samples<br>• Flexible calculation table<br>• Storage of planned and real reaction data<br>• Definition of starting material, reagents and product(s)<br>• Solvent management<br>• Standardized description modules available
Additional features & Background activities	External helpers	• OpenBabel<br>• NMRdb.org
	Supported external databases	• Pubchem<br>• SciFinder
	Calculations	• Automatic calculation of reaction table including yield<br>• Calculation according to flexible determination of reference material<br>• Calculation of expected molecular mass, exact mass and elemental analysis<br>• Transformation of temperature values from °C to °F to K
	External links	• Literature URL-management

Management & Organization	settings	• Editing of user profile<br>• Changing and update of credentials<br>• Definition of groups<br>• User defined reaction counter<br>• User defined labeling of samples/visibility
	Organization actions	• Delete, move to, assign to
	Search function	• Text search<br>• Structure search (exact search, similarity search, substructure search)
	Export and Import	• Export to Excel and SDF<br>• Import to Export and SDF
	Share data	• Share<br>• Synchronize
	Inheritance	• Sub-items (children) with inheritance of properties and information<br>• Clear and systematic labeling of sub-items and derivatives
	Codes and Tracking	• Barcode and QR-Code tracking
Technology in general	Languages and frameworks	• Ruby on Rails, Java Script: modern, easy to learn, intuitive
	Web-based	• Platform independent
	Open Source	• Adaptable, usable as stand-alone server installation

4. Procedures in pictures

4.1 Research management in collections

The traditional research management through the creation of distinct projects is reflected in the Chemotion ELN by so called collections (left panel, figure SI-1).The organization of the collections can be done in a separate management panel that allows a fast and easy labeling of the collections and their arrangement via drag and drop. Changes in the management UI are transferred to the collections organizer (left panel, permanently visible).

Figure SI-1. Management of collections as project planning and organization tool of the Chemotion-ELN: left: management of projects and visibility of connections; middle: extended view with subcollections; right: view within the ELN navigation bar.

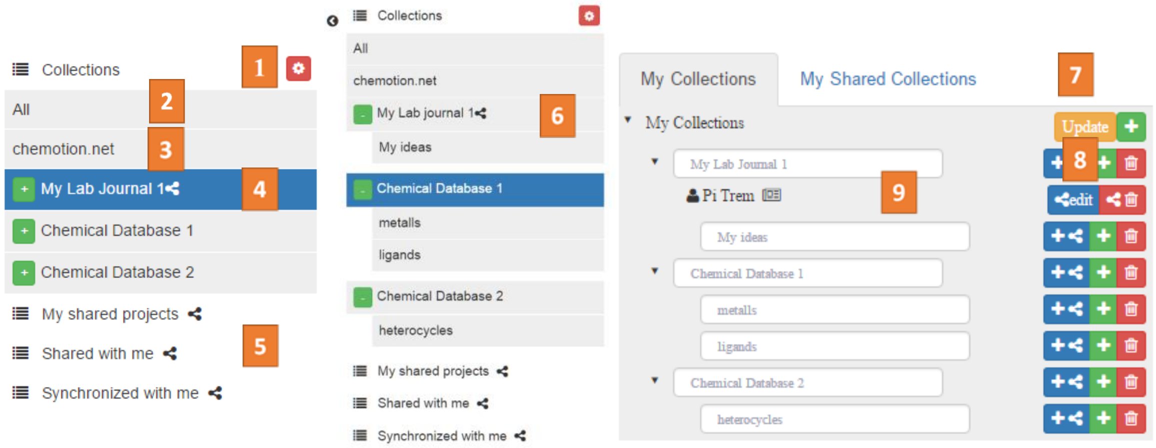

Legend:

1 The visible organization column (collection navigation) contains a gear icon button to access the collection management area where generation, editing and deletion of collections are done (see also info 7, right panel).

2 The standard collection *All* (standard collections are predefined collections for all users) contains all items. It functions as a backup service.

3 The chemotion.net collection (also a predefined standard collection for all users) is installed for a latter use in combination with a repository for chemical reactions and analyses.

4 Collections that contain sub-collections can be unfolded (see centered tab). Symbols indicate the collections which have been shared with others.

5 A separate organizer has been created for collections that have been shared, that are shared with the user (by others).

6 The tab in the middle of the figure shows the extended view of the organizer if collections contain nested collections (sub-collection). A deeper nesting of collections is also possible (not shown).

7 The management UI for the collections is divided into a management for collections of the user and collections that the user shared with others. Both management areas can be modified via drag & drop allowing the fast rearrangement of the single collections and the modification of the assignment.

8 Collections, subcollections and so on can be created, edited, extended and deleted at any time.

9 Additional information on the collection synchronization status with others are summarized below the collection entry. The assignment as asynchronized collection can be easily deleted or modified via the icons.

4.2 Organization of single elements and listing

Figure SI-2. Organization of elements (samples and reactions) in lists. Left: A selected list for samples with annotations for additional information. Right:A list of reactions.

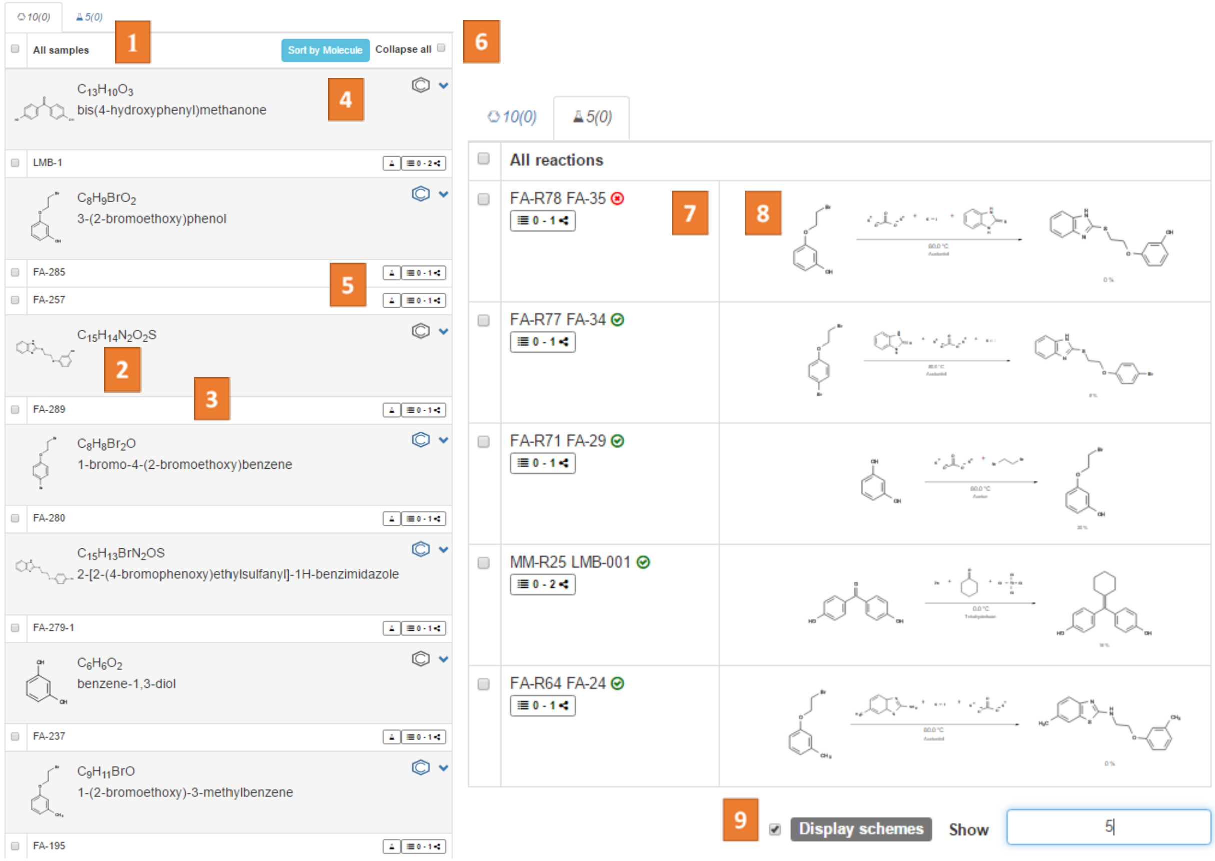

Legend:

1. Samples and reactions are summarized in lists which belong to particular collections. One can easily switch from the sample to the reaction view. One can select either single samples, whole pages, or the whole list of samples for actions (e.g. move/assign/share/copy etc.). The list can be sorted according to the date of modification or the molecular structure.
2. In sample lists, the items are sorted according to their molecular structure. Each sample is assigned to a molecule, one molecule can regroup several samples.
3. Samples are always listed below the assigned molecules and are represented by the molecular structure, the sum formula and the name of the compound (if available via Pubchem).
4. Molecules are automatically checked for their presence in the Pubchem database and the information on the presence or absence is summarized in the list. A grey-colored

Pubchem icon stands for “unknown in Pubchem”, a blue-colored icon means the structure is registered in Pubchem. In the latter case, the PubChem symbol serves as a link to the landing page of the molecule at the Pubchem website.

5 Additional information on the listed samples are summarized in information indicators. These small tags include information on the presence of the sample in other collections of the ELN, the sharing of the sample with others and the availability of analytical data that have been added to the sample and confirmed.

6 For a lighter view of the item list, it is possible to collapse all samples (showing only the molecules) or to collapse only selected list items.

7 Reactions are listed in reverse order of their reaction number (to let the most recent item appearing on the top). The reactions are given with the automatically created number of the reaction (including the initials of the user), the information on the sharing status and a symbol showing if the reaction has been successful or not.

8 The schemes of the reactions are listed.

9 The user may decide whether to display the schemes for the reactions. Other view adaptions are possible as well: the number of listed items per page can be adapted to the user needs (this feature works for samples and reactions).

4.3. Detail level for samples

Figure SI-3A. Detailed view of a properties tab including information of molecule and sample properties.

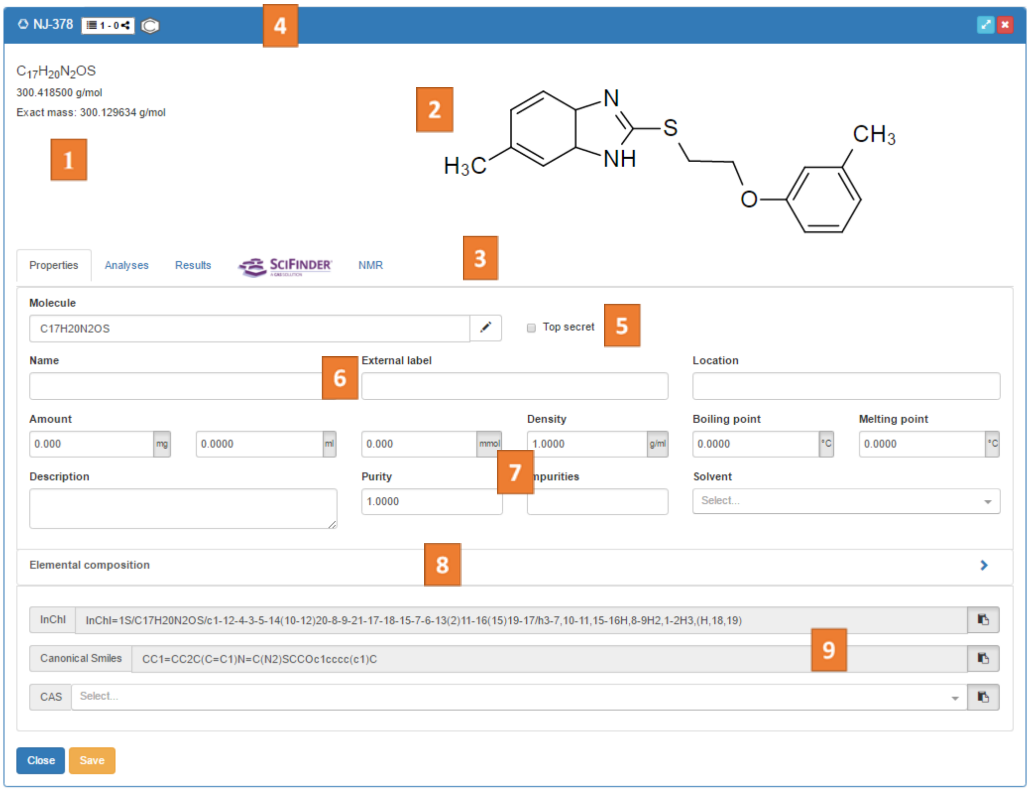

Legend:

1 The detailed view encompasses additional data that are available either through manual input or automatic request to external sources. The main data include the sum formula, the chemical name (IUPAC name), the molecular mass and the exact mass of the compound.

2 The structure of a molecule is given in a prominent manner and is visible independent of the selection of other tabs. Via simple click on the molecule, the structure editor can be opened allowing the modification of the molecule.

3 The details view combines the information of a molecule and the sample. The combined information is summarized in the three tabs properties, analyses and results. Additional data are available via the tabs SciFinder and NMR which are implemented as plugin

4 The window of the details tab is surrounded by a functional frame. The frame color switches from deep blue to light blue if unsaved changes have been made on the client side. If all information are saved on the server side, the frame changes to deep blue

again. In addition, the frame contains information on the name of the sample, its function in reactions that have been planned with the ELN (link to the reaction) and the share status of the sample.

5 The user can define a name for the sample and the sample can be defined as “top secret”. The latter status prevents the information of the sample from being shared and distributed.

6 While the molecule name of a structure is retrieved from Pubchem if possible, the name of the sample can be changed by the user. A secondary name and a location can be inputted as well.

7 Additional data such as the amount of the sample (in mg, ml and mmol), its density, the boiling point and melting point can be entered The details window summarizes in addition a description (textual), the purity (number) of the compound, impurities (textual) and a solvent (for a dissolved sample, selection via dropdown). The field purity affects the amount of the sample in mmol for a given mass.

8 A collapsible panel also contains information about the calculated elemental analysis. The user may use the form to fill the experimentally found elemental analysis data.

9 Selected Identifiers like the InCHI and Smiles Code are given to allow a fast search of the molecules in other databases: the identifiers can be copied to the clipboard with a single click. Additionally, the CAS registry number is given for those compounds that are available via PubChem (see Figure 3B).

Figure SI-3B. Connecting the retrieved CAS registry numbers with the samples (example: benzene).

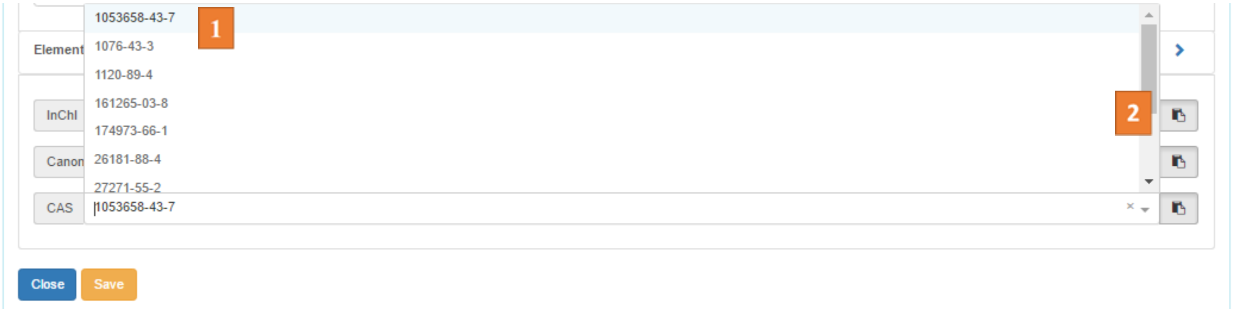

Every sample is assigned automatically to a molecule, and so to an InChI and SMILES identifier generated with OpenBabel. The InChI key is used to request the CAS registry number *via* the PubChem API service. The answer set is listed in a dropdown menu allowing the user to select the correct CAS RN. The answer set of the PubChem service includes CAS RNs for the requested InChI key and known isotopic derivatives of the requested InChI key. As the PubChem response also contains in some cases CAS RNs that belong to different molecules, forthcoming developments will provide an additional query alternative for double checking of the returned CAS RNs.

Legend:

1 If CAS registry numbers for a molecular structure are retrievable via PubChem, the available CAS numbers are stored with the molecule. The user can assign one CAS number to a particular sample by choosing from a dropdown menu.

2 All identifiers can be copied to the clipboard.

Figure SI-3C. Detailed view of a properties tab including information of molecule and sample properties. View of the analysis tab of the given structure.

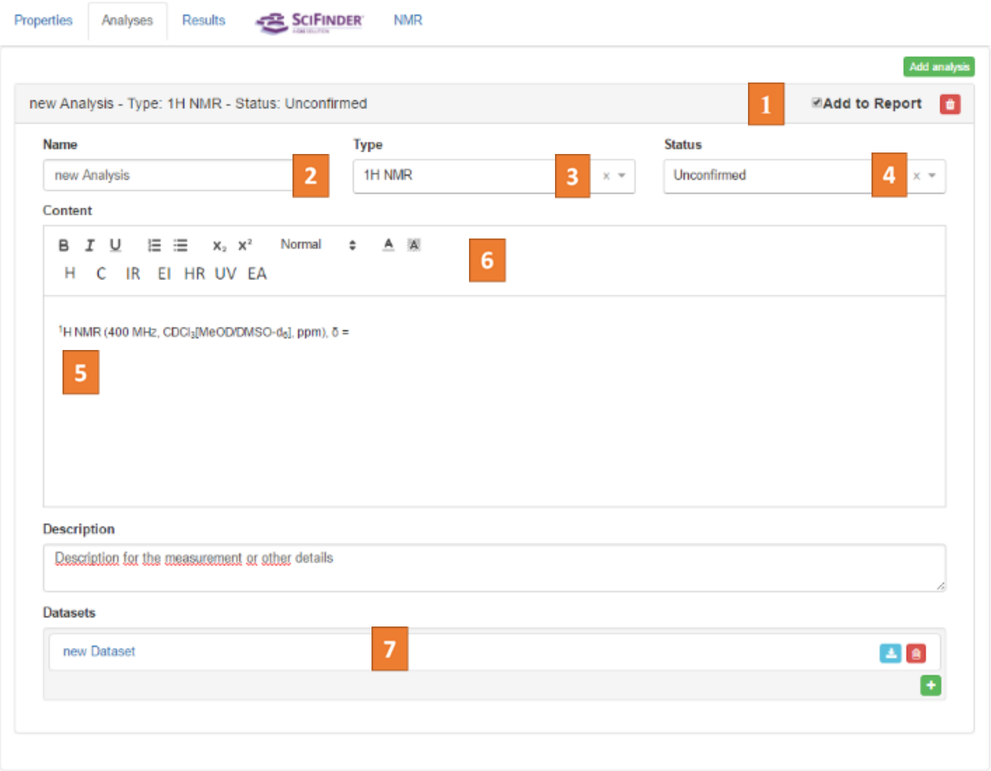

Legend:

1 The Analysis Tab allow the addition of one or more analyses. The user may select, whether the data are relevant to be included in reports.

2 The user can define a name for the analyses (textual).

3 The type of the analysis can be selected from predefined values from a dropdown menu to keep a standard classification.

4 The status of the analyses can be selected (confirmed, unconfirmed), the total count is indicated for each sample in the list view. Only the confirmed analyses are counted.

5 The free text field named *content* can be used for the textual summary of the analytical result.

6 To allow a fast and standardized text input for the content of the analysis, several icons have been created allowing the introduction of basic text parts that are necessary for the description of the standard analytical methods. In addition, text formatting functions are available.

7 For each analysis, several datasets can be attached. Datasets are uploaded and stored on the local server and can be retrieved via download.

Figure SI-3D. Upload of files.

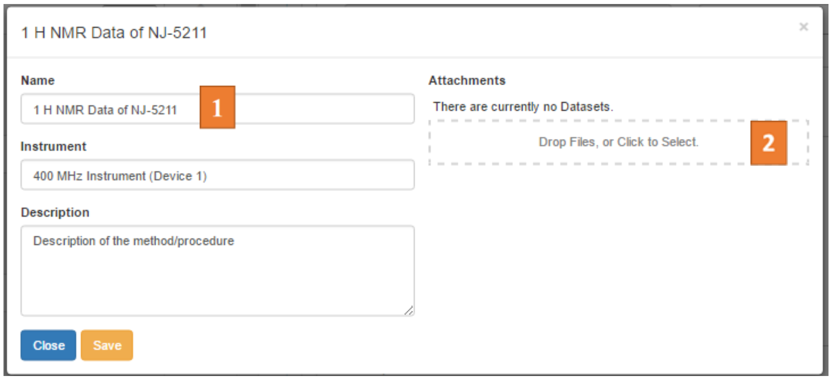

Legend:

1 Datasets belonging to the corresponding analysis can be named individually and details for the instrument as well as a general description of the processing method that been used can be given.

2 The attachments can be uploaded from any available storage or can be dropped into the datasets area.

Figure SI-3E. Results Tab.

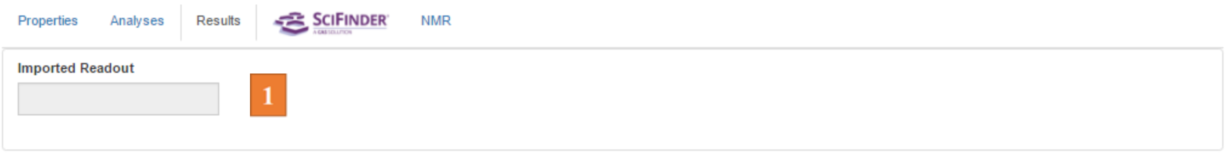

1 The results tab stores information that has been gained from external sources e.g. a collaborator that provides additional data. The textual field can be filled automatically from Excel sheets with a suitable formatting.

4.4 SciFinder Credential and requests

Figure SI-4. Left: Changing the user settings with the SciFinder credentials to obtain a user token with time-limitation. Right: SciFinder Tab with results of a database request with 4 hits identified for the exact structure search.

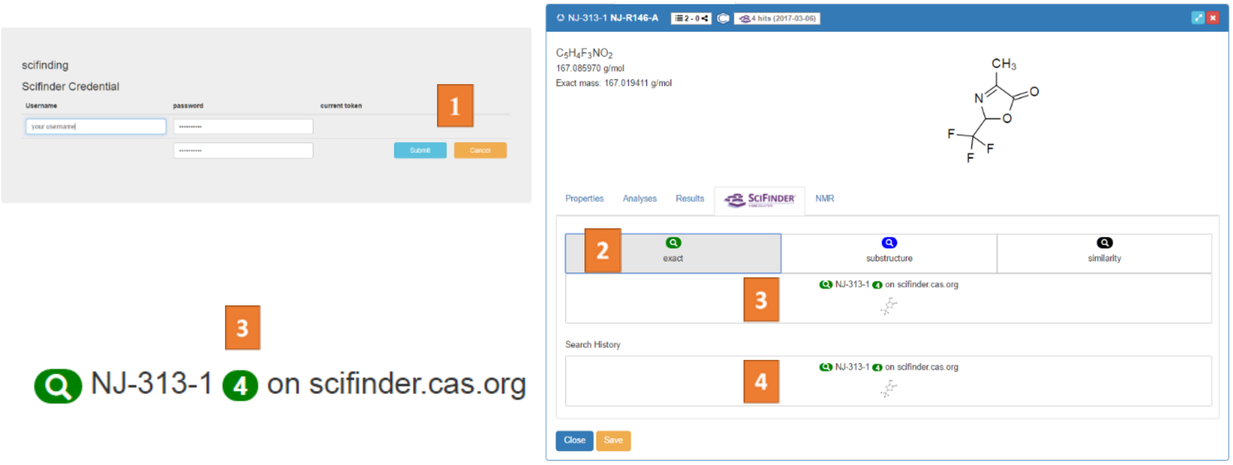

Legend:

1 To use the SciFinder Plugin, the user has to enter the SciFinder credentials. This procedure has to be repeated once the token has expired.

2 The search functionality allows the same search structure as the SciFinder online application, the user can chose between exact, substructure or similarity search.

3 All results of the search request are listed and the information is provided via a direct link to the search result on the SciFinder web page.

4 In parallel to the search, recent search results for the actual session are listed in a search history

4.5 Listing of database requests

Figure SI-5. Request for presence and accessibility of information to specific molecules via PubChem and embedding of the answer sets in Chemotion ELN.

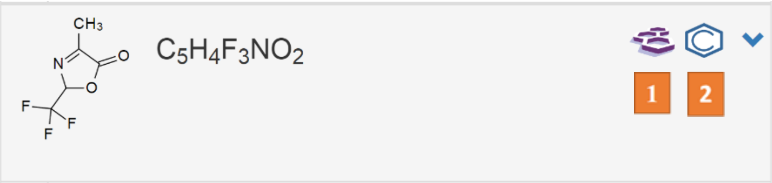

Legend:

1 The search results that have been gained via the SciFinder search are visualized in the list of the samples. Upon hovering over the SciFinder symbol, the date of the database request and the given results are listed (not shown).

2 The list format includes also information about the availability of Pubchem data related to the sample. In opposite to the search for the structure in Pubchem, no user request is necessary and the update of the information in the ELN is done automatically. For the PubChem request, only the exact search function is used.

4.6. Planning and editing reactions

Figure SI-6A. Planning and editing reactions via the Chemotion ELN: direct connectivity to sample related data.

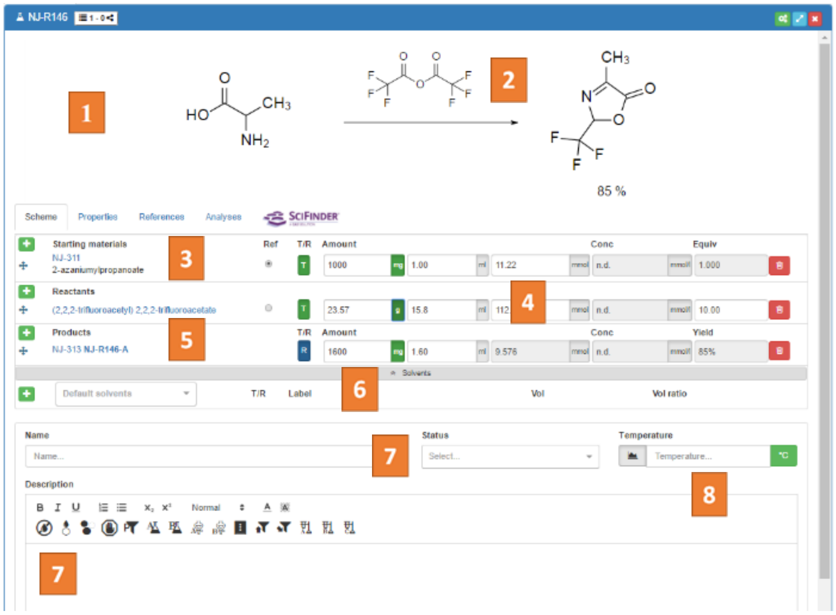

Legend:

1 The reaction scheme image can be shifted or zoomed in through mouse action.

2 Reagents are always given above the reaction arrow. All items of the reaction scheme like starting material, reagents, and product(s) are linked to the samples entries (shown exemplarily in the manuscript).

3 All samples that are given in the reaction table are given with the systematic ID of the ELN and additional external names and identifiers if possible. New samples can be submitted to the reaction (to any role) either by drag-and-dropping from the samples/molecules list or by drawing the structure with the editor.

4 All values are calculated according to the input of the user. Units are switchable and the user may select between the planned amount and the real amount that has been used after weighing of the materials. The reference compound is always set to be 1 equiv. per definition and can be switched in an easy manner. The definition of the role of a sample within the table can be changed as well (e.g. from reagent to starting material).

5 Products and calculations with material that occurs as a product differ from reagents and staring materials. The numbering of the product depends on the reaction name and the different products or fractions are labeled with the reaction ID and the letters (e.g. from A to D). The automatic calculation of the yield out of the outcome of the reaction is implemented while the direct entry of the yield without knowing the obtained mass is disabled to avoid misuse of the ELN.

6 Solvents can be added either by a drag-and-drop from the samples list, by the generation of a structure or by choosing one from a standard solvent dropdown menu. The amount of solvent is used to define the concentration of reagents in the reaction mixture.

7 The description of the reaction can be given and created individually or one can use pre-defined text blocks for a fast standardized entry (see Figure SI-6B).

8 The temperature of a reaction can be entered either as a number or can be given in a detailed way via creating an online temperature chart (not shown)

Figure SI-6B. Adding narrative information to the description of a reaction.

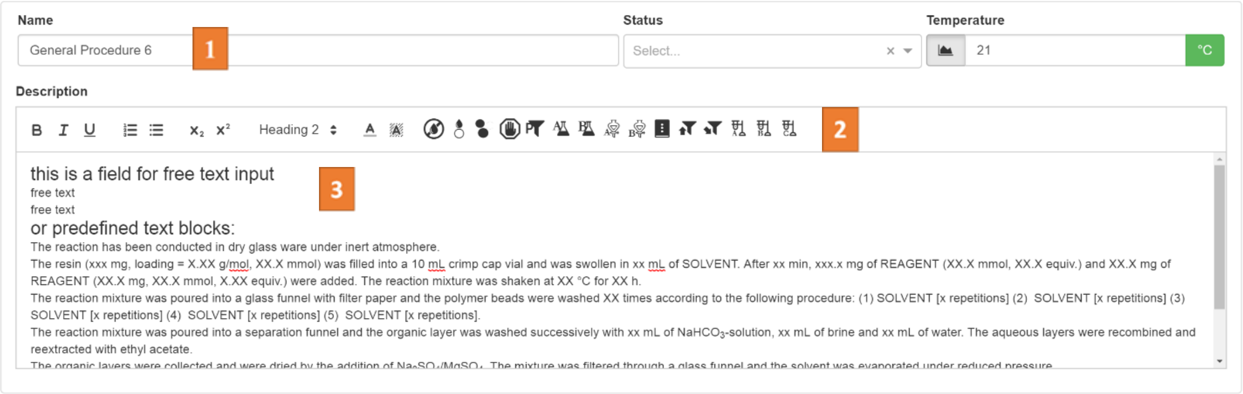

Legend:

1 The name of the reaction can be entered as free text by the user; the status can be chosen according to planned, successful and not successful and the temperature can be entered either as one value or a period (represented as a diagram)

2 There are at the moment 15 predefined text blocks that can be inserted for a fast standardized reporting on common procedures. The blocks can be combined to describe the whole synthetic process (if suitable for the reaction).

3 The description input field combines the free text input and the predefined text blocks. The text can be edited and modified according to the user's preferences.

Figure SI-6C. Adding narrative information to the description of a reaction.

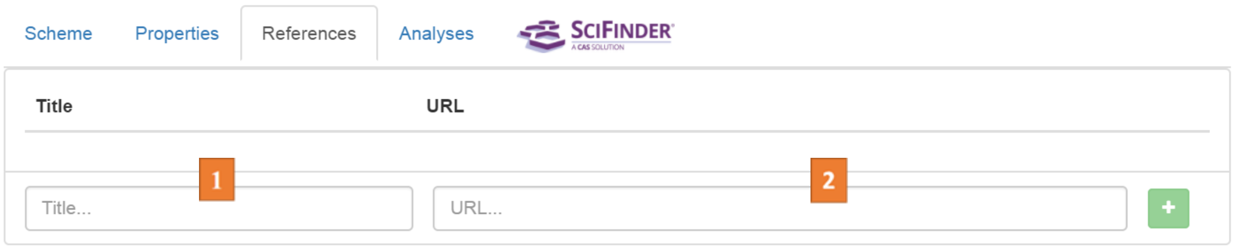

The ELN in its current version allows the input of information on references that are important or should be cited along with a reaction. At this stage, the ELN has a limited function to register a title, along with a URL. This will be superseded soon by a more advanced reference system linking Zotero citation management accounts..

Legend:

1 A new reference is added to a reaction directly via a tab that can be opened in the reaction area of the user interface. The user can add a title to structure the entries.

2 The URL of the reference can be entered and stored for a fast retrieval of the original text. The input field supports the addition of several references to one reaction.

4.7 Configuration of export functions and results (examples)

Figure SI-7. Export scheme allowing the selection of single items to be exported to the xslx or sd file format.

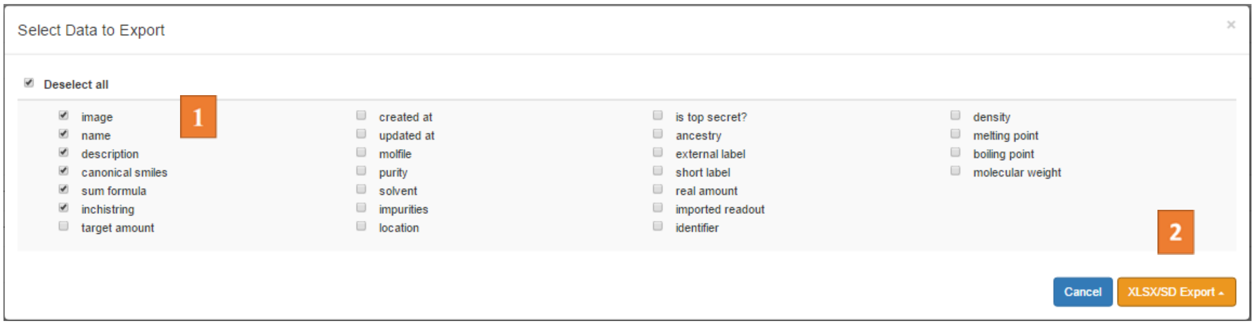

Legend:

1 The user defined export of data is managed via a list of possible data that can be included into the export file. The following information can be selected: image (image of the sample), name (user defined name of the sample), description (description of the molecule), cano_smiles (canonical smiles code), sum_formula (sum formula of the molecule), inchistring (InChI string), target amount (amount that was planned), created_at (date-time of creation), updated_at (date-time of last update), molfile (molfile of the compound), purity (purity), solvent (for diluted samples: solvent), impurities (textual, qualitative information on impurities), location (location of the sample), is_top_secret (Boolean describing sensitive information), ancestry (hierarchical sequence of sample ids), external_label (additional label provided by others or to provide for others), short_label (automatically generated label for samples), real_amount (amount that has been effectively used), imported readout (data obtained from external sources), identifier (InChIKey, molecule identifier, density (density), melting_point (melting_point), and boiling point (boiling point), and molecular weight (molecular weight).

2 The export can generate either a XLSX file or an Sd file

4.8 Sharing of information (single user and group level)

The sharing of information can be managed via the selection of a single user or a user group. The user groups have to be defined by each ELN user individually.

Figure SI-8. Left: Creation of new user groups; Right: Definition of user groups and assignment of sharing role, permission level and available detail level for single users and user groups.

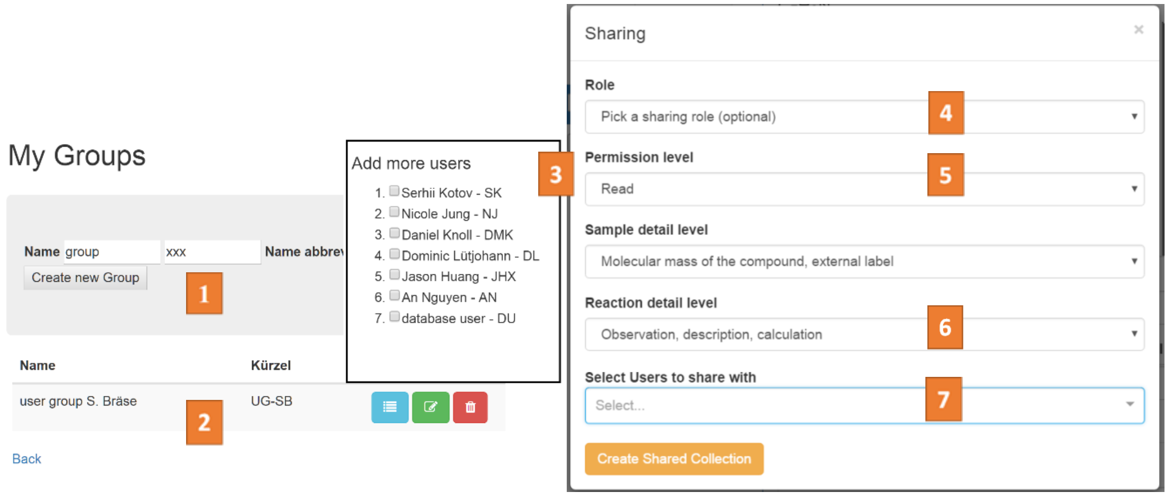

Legend:

1 The creation of a new user group (with a short label for the group) can be managed via the user settings in the ELN. The user can define as many groups as necessary and can chose the members of the groups

2 The user groups can be deleted, edited and the colleagues that are part of the group can be listed.

3 New users are added via selection form a list of all ELN users

4 The sharing roles allow a fast assignment of the rights (read, write, share, delete, import elements, take_ownership) to a colleague or a group. The read level is the lowest level of rights, the take_ownership level is the highest one. The fast assignment includes also a predefined detail level that is available for samples or reactions.

5 The permission level can be assigned either through the sharing role or can be set individually.

6 Sample and reaction detail level can be chosen individually or given automatically via the sharing role. There are two levels for reactions (level 1: Observation, description, calculation; level 2: everything) and 4 levels for samples (level 1: molecular mass of the compound and external level; level 2: molecule, structure; level 3: analysis result and description; level 4: analysis datasets, level 5: everything).

7 In the last step, colleagues are selected from the list of users of the ELN.

4.9. Search functions

Figure SI-9A. Search functions using the refined search function of the ELN.

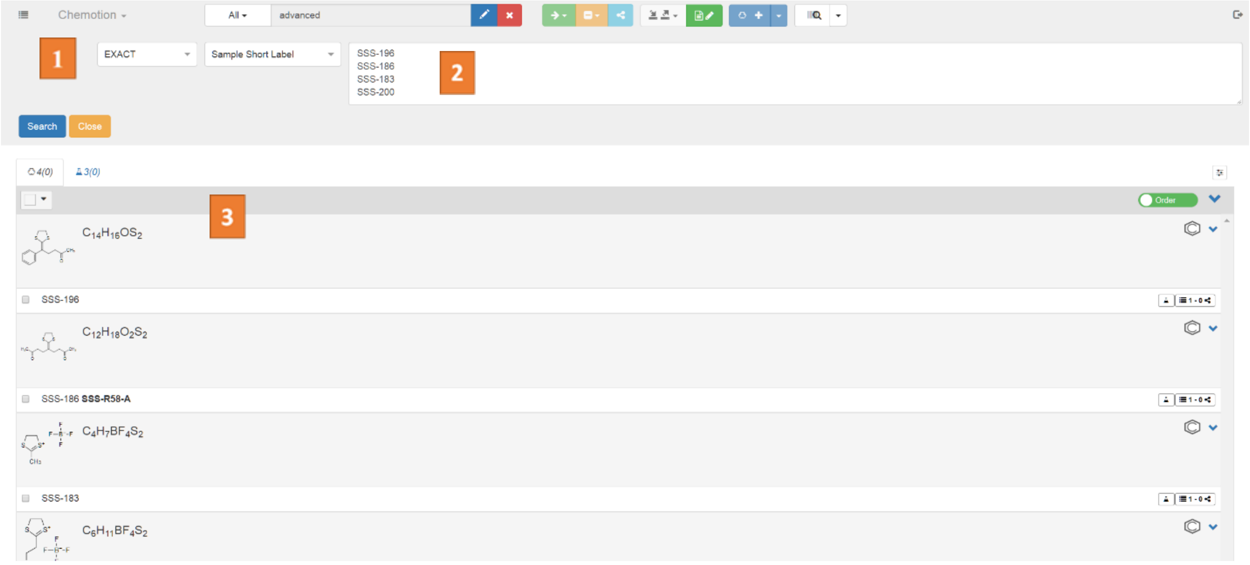

Legend:

1 The contents of the ELN can be searched by a text search. The user can switch from a simple search mode (searching for one text fragment in all items) to a refined search mode. The refined search mode supports *EXACT* and *SUBSTRING* search and currently the search in four different ELN areas (example in Figure SI-9A is *sample short label*)

2 The user may input either single search items or a set of items to retrieve a list of compounds as a result of the search function.

3 The results are listed according to the order of the original entry

SI-9B. Search functions with structure and substructure search (adaptable through similarity search).

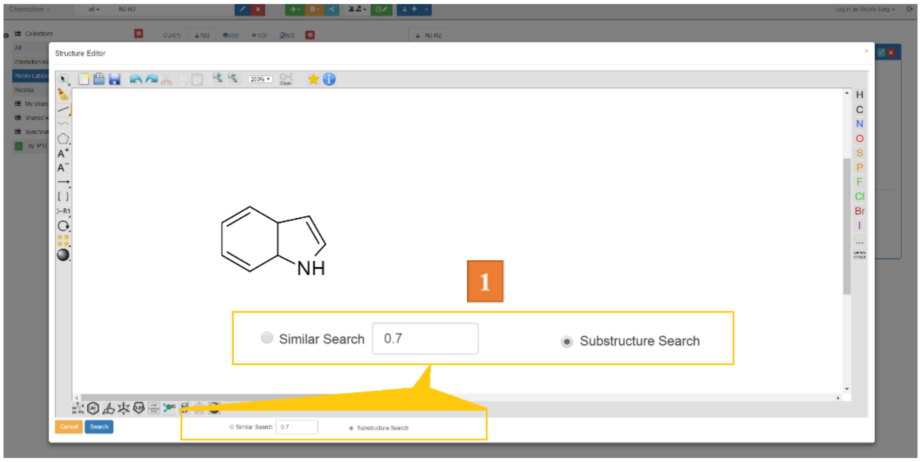

Legend:

1 The contents of the ELN can be searched by a text based search and a structure search. The structure search can be adapted to the user's needs by choosing either the similar search (and defining the degree of similarity) or the substructure search.

4.10. Coding and Tracking

Details on the used QR Codes and BarCodes:

Samples, Reactions, and Analyses objects are assigned a UUID v4. The code is translated into a 40 digit sequence and can be displayed as QR code (version 1 data correction level L or version 2 data correction level Q), or truncated (10 digits) Barcode of type 128C.

Figure SI-10A. BarCode and QR code generation, printing and tracking of samples via code reading.

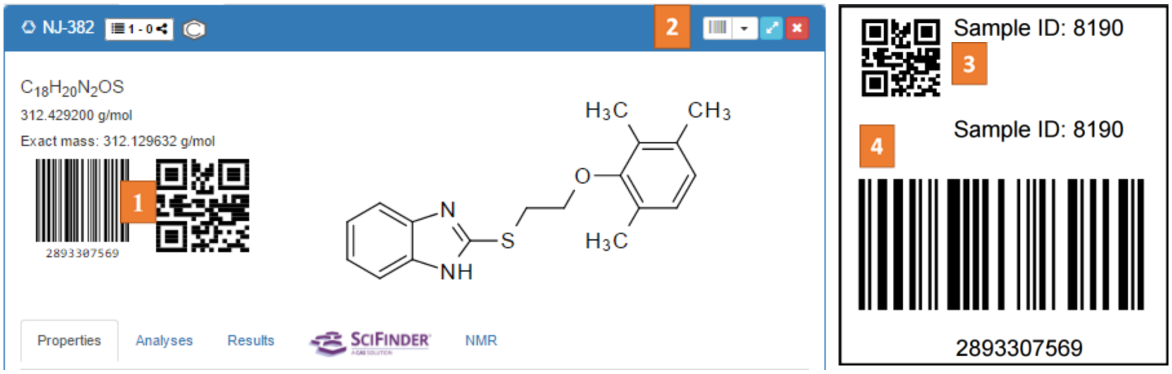

Legend:

1 Upon creation of a new item, a QR- and a Barcode are created for every sample and every new reaction. In addition, the QR- and Barcodes are generated for each analysis that is planned in the ELN.

2 Barcodes and QR-codes can be printed easily. As of now, two sizes are offered to meet the requirements of bottle labeling and sample labeling for GC-vials or PCR tubes. The labels are generated as pdf (see description of 3 and 4) and can be printed with e.g. on label printer (used in the demo version in the KIT labs).

3 The printed label contains one QR- and one Barcode assigned to the sample/reaction/analysis.

4 The additionally printed Sample ID to facilitates the assignment of the label to the item that should be labeled

Figure SI-10B. Barcode and QR-code reading via PC-lens or external WebCam.

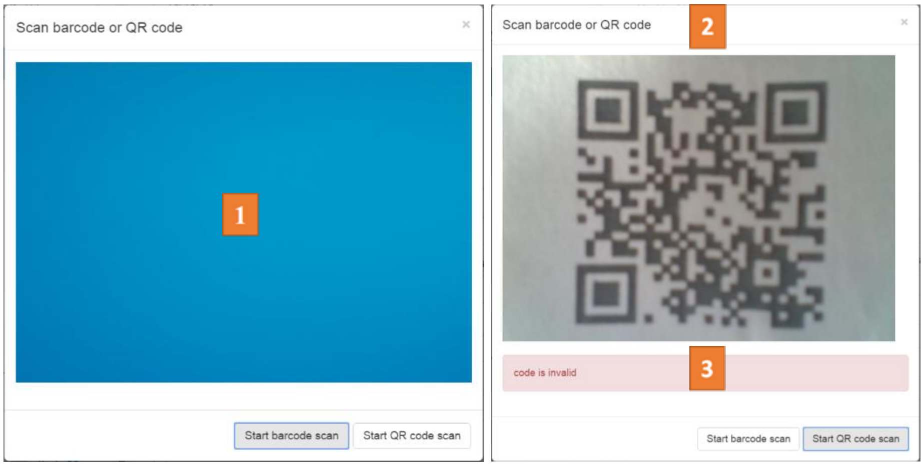

Legend:

1 Starting of the webcam function to read the BarCode or QR-Code

2 Scanning of a QR Code as an example.

3 QR code taken from an external source that can't be recognized by the ELN. Valid Code would initiate a direct link to the registered item.

Alternatively, code scanning devices which are recognized as input devices (e.g. usb handheld 2D code scanner), can also be used.
